# SurvivEHR: a competing risks, time-to-event foundation model for multiple long-term conditions from primary care electronic health records

**DOI:** 10.1038/s41746-026-02709-z

**Published:** 2026-05-09

**Authors:** Charles Gadd, Krishna Gokhale, Aditya Acharya, Jennifer Cooper, Francesca Crowe, Leah Fitzsimmons, Thomas Jackson, Krishnarajah Nirantharakumar, Christopher Yau, The OPTIMAL Consortium, Rebecca Birch, Marco Canducci, Dominic Danks, Alexander d’Elia, Alastair Denniston, Sarah Flanagan, Suzy Gallier, Naijie Guan, Xin Guan, Imane Guellil, Georgios Gkoutos, Shamil Haroon, Eleanor Hathaway, Louise Jackson, Janet Lord, Zeinab Majid, Tom Marshall, George Morris, Charlotte Owen, Elizabeth Sapey, Chris Sainsbury, Charlotte Spurway, Peter Tino, Steven Wambua, Amaya Azcoaga-Lorenzo, Colin McCowan, Luciana Rocha Pedro, Muhammad Usman, Natalia Hong, Sara Matijevic, Kaspar Martens, Tim Williams, Puja Myles

**Affiliations:** 1 Nuffield Department for Women’s & Reproductive Health, University of Oxford, Oxford, UK; 2 University of Birmingham, Birmingham, UK; 3 Kings College London, London, UK; 4 Health Data Research UK, London, UK; 5 University of St Andrews, St Andrews, UK; 6 Medicines and Healthcare products Regulatory Agency, London, UK

**Keywords:** Computational biology and bioinformatics, Diseases, Health care, Mathematics and computing, Medical research

## Abstract

Multiple long-term conditions (MLTCs), or multimorbidity—the co-occurrence of multiple chronic conditions—present a growing challenge for primary care. Existing predictive models typically focus on single outcomes and often fail to capture the temporal and competing-risk structure inherent in longitudinal electronic health records (EHRs). Here, we present SurvivEHR, a generative transformer-based foundation model trained on over 7.6 billion coded events from 23 million patients in UK primary care. SurvivEHR is pre-trained using a competing-risk, time-to-next-event objective, enabling calibrated risk stratification across a broad range of diagnoses, investigations, medications, and mortality events. We show that this pre-training objective yields strong next-event discrimination and learns clinically meaningful patient trajectories. When adapted through fine-tuning, SurvivEHR achieves improved performance on downstream prognostic tasks, including longer-horizon risk prediction, with particular benefits in low-resource settings. By learning longitudinal patient representations directly from routine primary care records, SurvivEHR provides a scalable foundation for developing generalisable clinical risk models that reflect the complexity of MLTCs in primary care.

## Introduction

The study of multiple long-term conditions (MLTCs), also referred to as multimorbidity, is becoming increasingly important as the prevalence of individuals living with two or more chronic conditions continues to rise^1^. This shift is largely driven by an ageing population and advances in medical care that have extended life expectancy, resulting in more people living longer with chronic diseases^2^. MLTCs are now the norm rather than the exception in many healthcare settings, particularly in high-income countries, and are associated with poorer health outcomes, reduced quality of life, increased healthcare costs, and higher rates of hospitalisation and mortality^3,4^. Despite the scale of the issue, healthcare systems, research frameworks, and clinical guidelines often remain structured around single-disease models, which do not reflect the complex realities faced by patients with MLTCs^5^.

In many health systems, primary care plays a central role in the management of MLTCs, serving as the first point of contact for most patients and providing continuous, coordinated care across multiple specialities. General practitioners (GPs) are uniquely positioned to understand the broader context of a patient’s health, including the interplay between physical, mental, and social factors. However, the growing complexity of care required by patients with MLTCs places significant strain on primary care services and exposes gaps in the evidence base that underpins clinical decision-making^6^. Effective management of MLTCs in primary care requires a shift away from disease-specific approaches toward more holistic, patient-centred models that consider the cumulative and interactive effects of multiple conditions^5^.

Many current clinical risk prediction models that are in use remain focused on single conditions, such as cardiovascular disease (CVD)^7,8^, diabetes^9^, or cancer^10^. While these models can be useful in targeting disease-specific interventions, they are not designed to account for the wider health status of patients and, in fact, often only accept input of the patient’s current characteristics. This may lead to missed opportunities for improved care and prevention^2^ and there is a need to develop and implement risk prediction tools that better reflect the complexity of MLTCs, particularly in primary care, where early identification and proactive management can be most effectively implemented.

Deep learning (DL)-based models for electronic health records (EHRs)^11^ have enabled a new generation of EHR-based predictive models that overcome the limitations of traditional risk prediction models. Predictive models using cross-sectional (non-longitudinal) inputs such as Deephit^12^ and DeSurv^13^ can provide competing risk, time-to-event models to flexibly handle heterogeneous input features. However, models that specifically utilise longitudinal inputs are capable of learning from and using entire patient histories, thus potentially accounting for complex MLTC backgrounds (Table 1). Recently, these EHR-based *foundation models* have been developed, which learn general patterns from EHR data that can be used as a basis for specific prediction tasks via transfer learning or direct zero-shot prediction involving no fine-tuning or retraining^14^. These include Delphi-2M^15^, which was built using primary care data for 400,000 individuals from the UK Biobank^16^ and examines risks for over 1000 health conditions. Curiosity^17^ has been built using 16.3 billion encounters over 300 million unique patient records from 310 health systems using the Epic system. Methodologically, the closest to what we propose is MOTOR^18^, which was the first EHR foundation model that formulates time-to-event prediction as a multi-task, single-risk problem, where each outcome is treated as an independent survival task with its own prediction head and piecewise exponential likelihood.

**Table 1:** A summary of existing deep learning-based clinical risk prediction models

Style	Model	Event types				TTE	# Patients	
		Diagnoses	Investigations	Investigation results	Medications	Survival	Primary care focus	Total
Decoder-only	SurvivEHR	✓	✓	✓	✓	✓	22M	22M
	CoMET^17^	✓	✓	✓	✓	✗	✗	118M
	MOTOR^18^	✓	✓	✗	✓	✓	✗	2.6M
	Doctor-AI^72^	✓	✗	✗	✓	✗	0.3M	0.3M
	Delphi-2M^15^	✓	✗	✗	✗	✗	0.2M	0.5M
	ETHOS^58^	✓	✓	✓	✓	✗	✗	0.3M
	Foresight^41^	✓	✓	✗	✓	✗	✗	0.8M
	MedGPT^40^	✓	✗	✗	✗	✗	✗	0.6M
	Event-stream GPT^37^	✓	✓	✓	✓	✗	✗	0.3M
	MetaCare++^73^	✓	✗	✗	✗	✗	✗	0.0M
Contrastive	Hi-BERT^74^	✓	✓	✓	✓	✗	2.4M	2.4M
	OCP^75^	✗	✗	✗	✗	✗	✗	0.1M
Masking	T-BEHRT^76^	✓	✗	✗	✗	✗	6.8M	6.8M
	life2vec^38^	✓	✗	✗	✗	✗	3.3M	3.3M
	BEHRT^36^	✓	✗	✗	✗	✗	1.6M	1.6M
	Med-BERT^57^	✓	✗	✗	✗	✗	✗	20M
	TransformEHR^39^	✓	✗	✗	✗	✗	✗	6.5M
	CEHR-BERT^77^	✓	✓	✗	✓	✗	✗	2.4M
	CMS LDS BERT^78^	✓	✗	✗	✗	✗	✗	1.2M
	RAPT^79^	✗	✓	✓	✗	✗	✗	0.1M
	Graph-Transformer^80^	✗	✓	✓	✓	✗	✗	0.0M
	GRACE^81^	✗	✓	✓	✗	✗	✗	0.0M
	MTL GPR^82^	✓	✗	✗	✗	✗	✗	0.0M

TTE (time-to-event) refers to learning the full distribution of the time-to-event, rather than ignoring time or making only a point prediction. Information not made available is left blank.

However, despite the plethora of DL prediction models, limitations on data availability remain a challenge and have had unintentional consequences on the type of models developed. Many models have been developed using secondary and tertiary care datasets, especially MIMIC-IV^19^. At the time of writing, we are only aware of a small number of DL models have been developed with a focus on primary care data (we searched PubMed for relevant studies using the terms (‘primary care’ AND ‘deep learning’ AND ‘electronic health record’) and found only 12 total results of which most were not relevant) and these predominantly originate from European institutions that make use of European data sources such as the UK Clinical Practice Research Datalink (CPRD)^20^, Whole Systems Integrated Care Northwest London^21^, UK Biobank^16^, the Finnish Registry^22^, The Information System for Research in Primary Care (SIDIAP) in Spain^23,24^ and the Danish Health Registry data^25^. Primary care is represented in some U.S.-based health data resources, such as the PRIME Registry^26^, Veterans Health Administration data^27^ and in certain hospital network datasets, such as Stanford Medicine^28^, although coverage of primary care encounters within these sources can be variable and inconsistent. Proprietary medical insurance claims databases (e.g., Truven Health MarketScan and Partners For Kids) may indirectly capture some aspects of primary care through billing records rather than detailed clinical data. As a consequence, primary care data are closely shaped by local health system structures, clinical workflows, and data recording incentives (including reimbursement structures), their translation or interoperability across regional and national domains is often limited^29,30^. Models based on primary care data will therefore learn trajectories that represent a mixture of factors that may be very specific to a given healthcare system.

Motivated by the lack of models specifically targeting primary care, and informed by input from our patient advisory group, we propose a novel foundation model for time-to-event forecasting using UK primary care-based EHR data within a competing-risks framework that jointly accounts for MLTCs. Beyond predictive accuracy, this approach is intended to better reflect the complexity of real-world primary care, where clinicians must assess and prioritise risk across multiple interacting conditions rather than optimise for a single disease outcome. We hypothesised that pre-training on large-scale longitudinal primary care data would provide a platform for developing more robust and generalisable prediction models that support risk stratification and proactive care planning in complex patient populations. Based on the Generative Pre-trained Transformer (GPT) architecture^31^, we developed *SurvivEHR* and trained it on 7.6 billion coded events from 23 million patients in the UK CPRD^20^, demonstrating strong transfer learning performance across a range of downstream clinical prediction tasks.

## Results

### Model overview

We introduce *SurvivEHR*, a decoder-only transformer-based foundation model designed for next-event prediction with time-to-event estimation in longitudinal primary care EHRs. The model is trained in a self-supervised generative fashion, learning to predict both the type and timing of the next clinical event, thereby enabling calibrated temporal risk stratification across multiple dependent outcome types (competing risks).

**Fig. 1:**
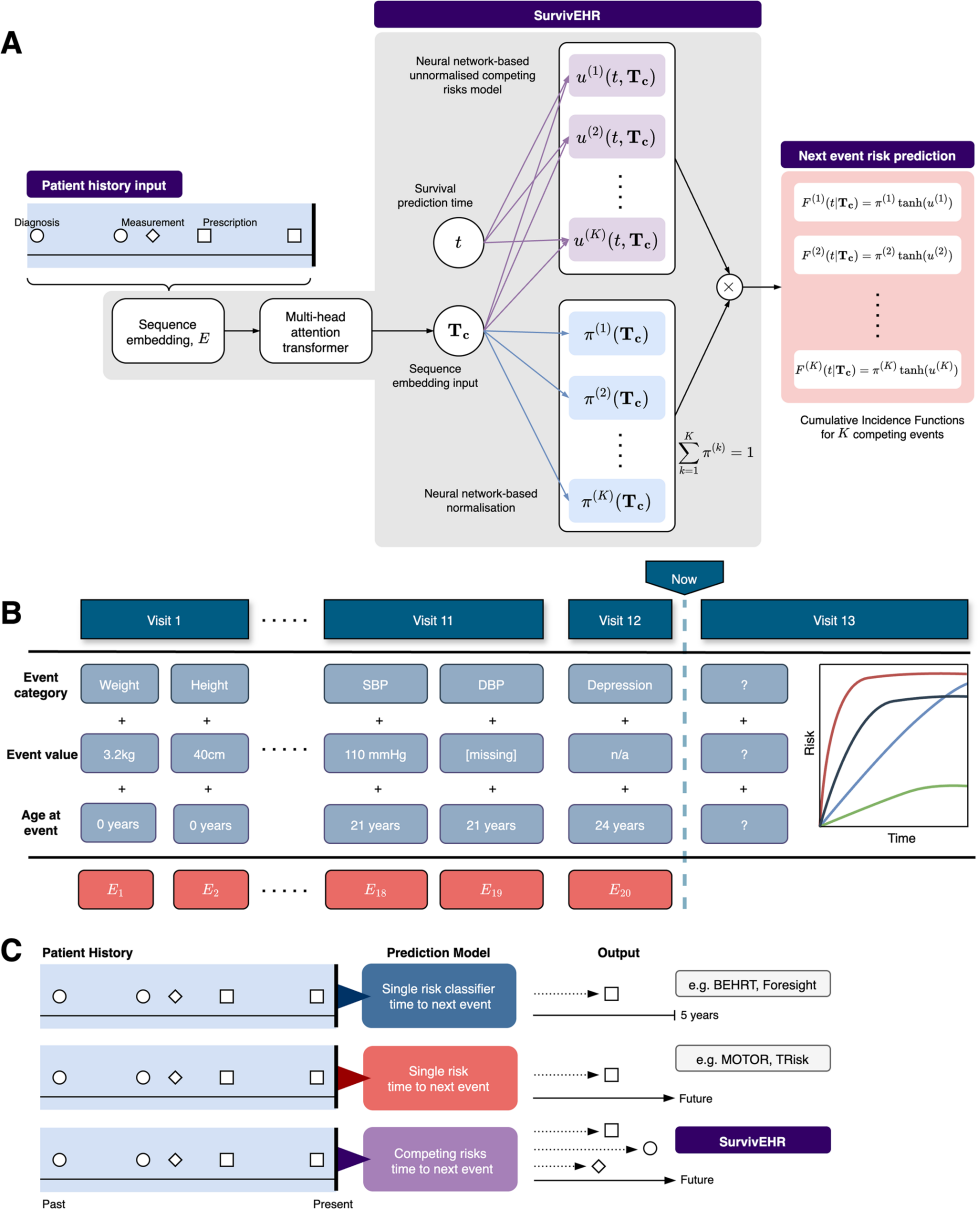
SurvivEHR. **A** Schematic overview of the SurvivEHR model architecture and implementation. Sequence embedding of tokenised patient histories is fed to a multi-head attention transformer, which is used within a neural network-based competing risk next-event prediction model. **B** Joint embeddings are created encoding event type, values and times to produce time-to-event competing risk and value prediction functions. **C** Competing risk time-to-event modelling distinguishes SurvivEHR from other related models that focus on discrete classification or single-risk modelling.

The input into SurvivEHR is a patient’s longitudinal history of clinical events—including diagnoses, prescriptions, and measurements—as a sequence of tokens (Fig. 1). Each token is embedded based on the event type, any associated numerical value (e.g. test result) and its timestamp relative to the patient’s age (Fig. 1B). This sequence is processed via a *multi-head attention transformer* producing a latent representation of the current patient state (Supplementary Fig. S1). To predict the time to the next event, SurvivEHR integrates a neural-based competing risks framework^13^ which yields calibrated cumulative incidence functions (CIFs) for each potential outcome, representing the risk that each event occurs before a certain time.

This distinguishes SurvivEHR from other DL EHR models in that its design is specifically catered to primary care and operates in a competing risk, time-to-event framework (Table 1). The approach is necessary since patterns of MLTCs in primary care are complex and diverse health events can occur over time scales across a person’s lifetime^32^. This makes the prediction problems different from those of predicting a single outcome risk, fixed period hospital readmission or disease recurrence and other problems which are well tackled by many existing DL EHR models.

### Data overview

**Fig. 2:**
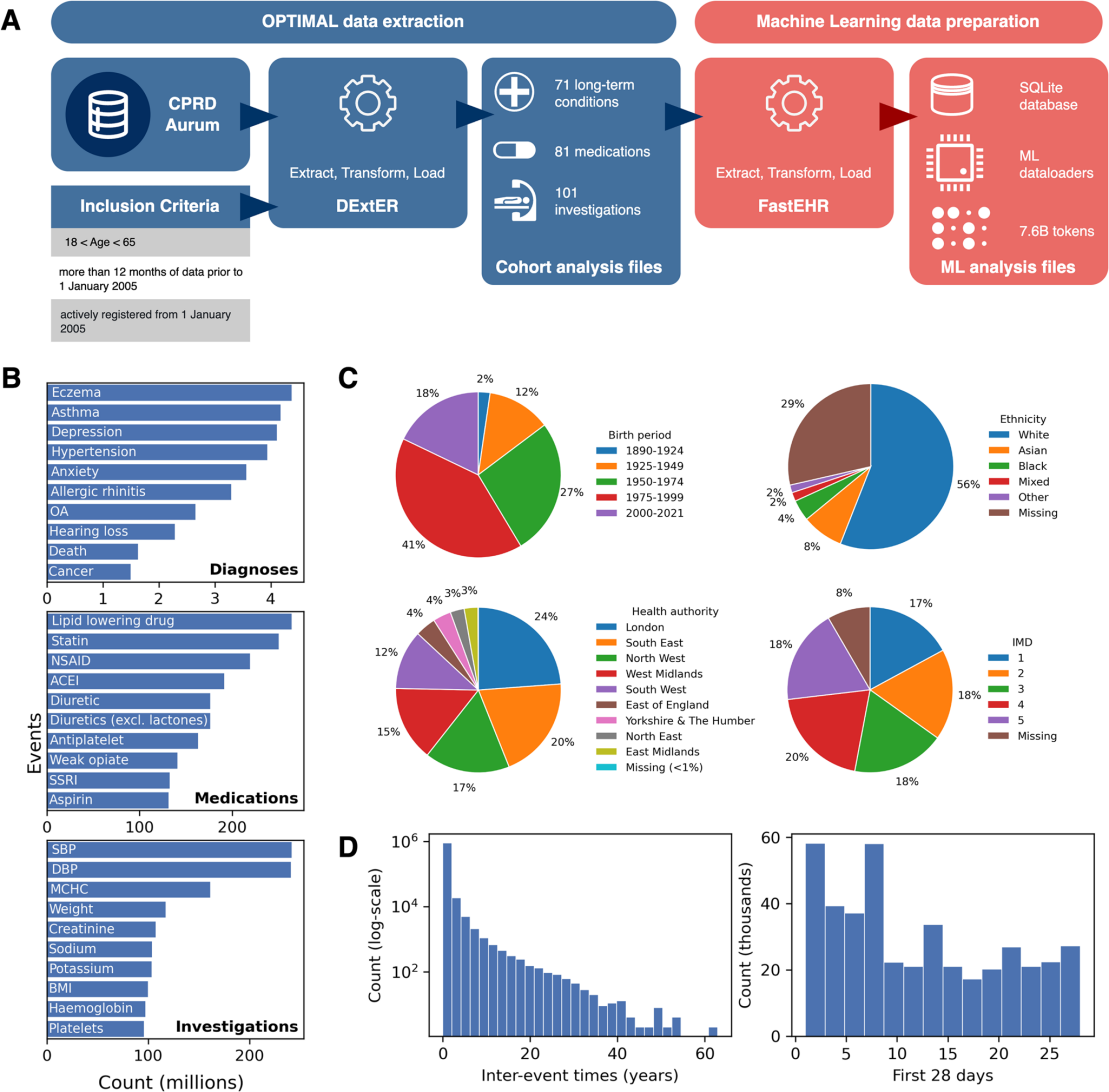
Overview of OPTIMAL cohort data preparation and content. **A** Data is extracted from CPRD Aurum using DExtER to produce a series of cohort analysis files. FastEHR is used to prepare the data for machine learning analysis. **B** Counts of the occurrence of different diagnoses, prescriptions and investigations. **C** Demographics summary of the individuals contained in the OPTIMAL cohort showing birth year, ethnicity, regional area and index of multiple deprivation (IMD). **D** Distribution of held-out inter-event times (left), all inter-event times (right), and inter-event times within the first 28 days. Figure created using draw.io.

The data processing pipeline is schematically depicted in Fig. 2A. We used the CPRD, a large longitudinal primary care database covering over 2000 UK general practices using EMIS Web®. Around 10 million currently registered patients and 30 million historical records are included, broadly representative of the UK population. CPRD access was granted via the ‘OPTIMising therapies, disease trajectories, and AI assisted clinical management for patients Living with complex multimorbidity’ (OPTIMAL) study.

Data were extracted using DExtER^33^. Diagnoses, medications, and investigations were recorded using Read V2, SNOMED-CT, and EMIS codes. Pre-processing was conducted using FastEHR, a Python toolkit for scalable, memory-efficient EHR pipelines, enabling tokenisation, filtering, and leakage control. Post-processing, we retained 51M diagnoses, 4B medications, and 3.5B test records, 3.4B with numerical values. We applied a 90-5-5 split by practice site to avoid leakage, resulting in 23.6M training, 1.4M validation, and 1.5M test patients.

**Fig. 3:**
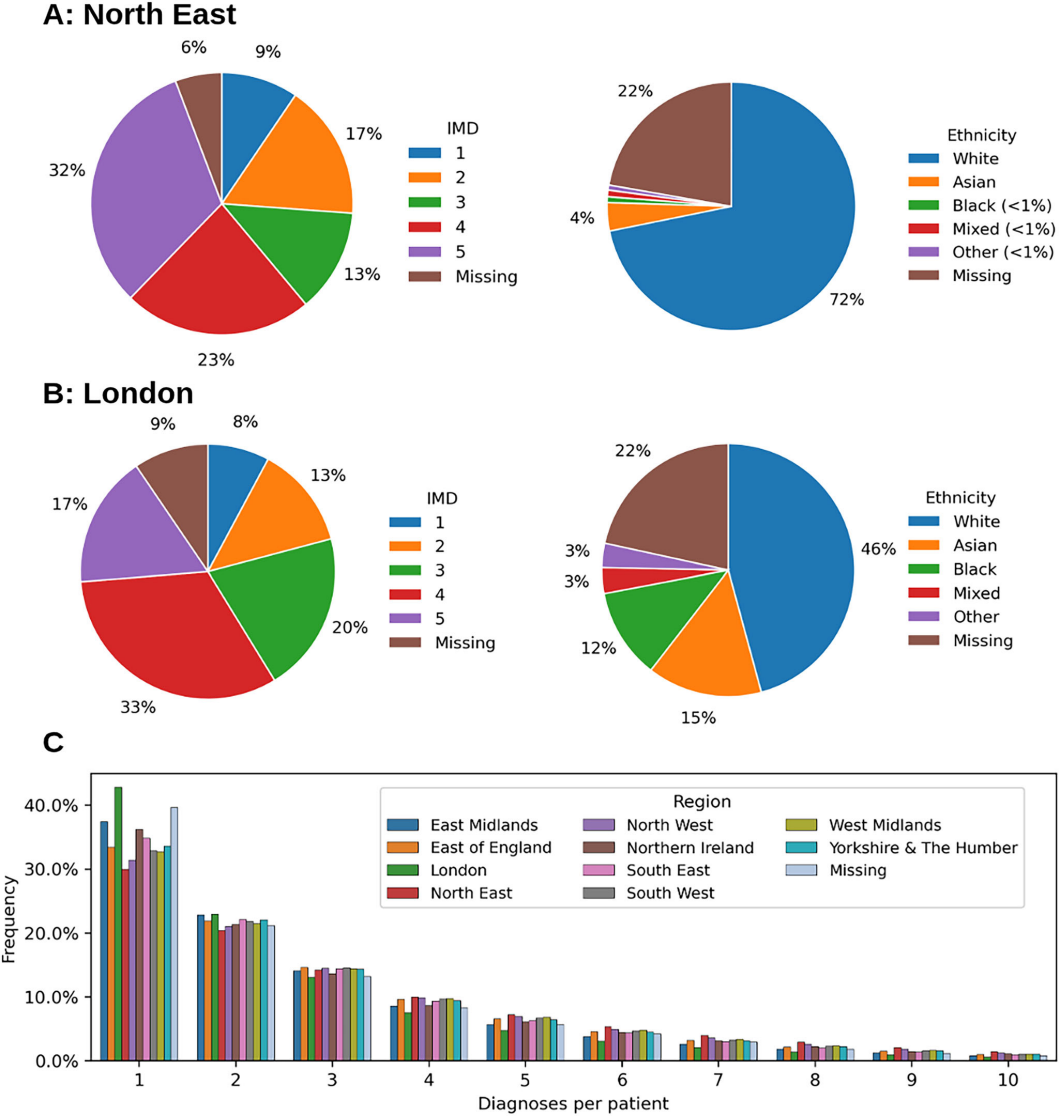
Regional shift of OPTIMAL cohort data. Index of multiple deprivation (IMD) and ethnicity summary of the individuals from **A** the North East of England, and **B** London, England, contained in the OPTIMAL cohort. **C** Distribution of multi-morbidity frequency between different regions in England.

This work focuses on MLTCs, and we identified 74 long-term conditions, 81 medication classes (based on DM&D Prodcodes), and 108 test types, including blood tests and physiological measurements. The most prevalent items are shown in Fig. 2B. Selection was informed by a Delphi study identifying 59 key conditions for multimorbidity research^34^ and refined through expert and patient advisory input^35^. We included English patients with at least 12 months of acceptable data before 1 January 2005, yielding 26.5 million patients from 1478 practices. The data covered a wide age, gender, ethnicity, geographical and index of multiple deprivation (IMD—a UK government-produced, area-level measure of socioeconomic deprivation) profile (Fig. 2C). As discussed previously, inter-event intervals spanned a range from days to years, which is consistent with lifelong patient history recording in primary care data (Fig. 2D, Supplementary Fig. S8). Figure 3 shows distributional differences in demographics between the North East of England and London and the number of diagnoses per patient by region.

### Next event prediction performance

The evaluation of the pre-training performance of a foundation model is a critical step in understanding its capabilities and limitations. Proper evaluation metrics allow us to examine how well the pre-trained model aligns with downstream application requirements and provide insights into its utility as a transferable learning framework. We evaluated the performance of SurvivEHR on its core task of risk-stratification for the next event given a patient history, introducing a new performance metric—the Inter-Event Concordance (IEC) metric—to assess risk discrimination in self-supervised survival modelling (see ‘Methods’).

We compare our pre-training strategy with two alternatives. Firstly, we consider an entirely prevalence-based risk policy as a baseline, in which we prognosticate with a risk proportional to an event’s prevalence in the population. Secondly, we consider a benchmark foundation model built upon the same backbone GPT architecture, but using a multi-label classification (cross-entropy based) objective frequently used in clinical foundation models^36–41^. For this approach, we assign the risk to be proportional to the classification probability.

**Fig. 4:**
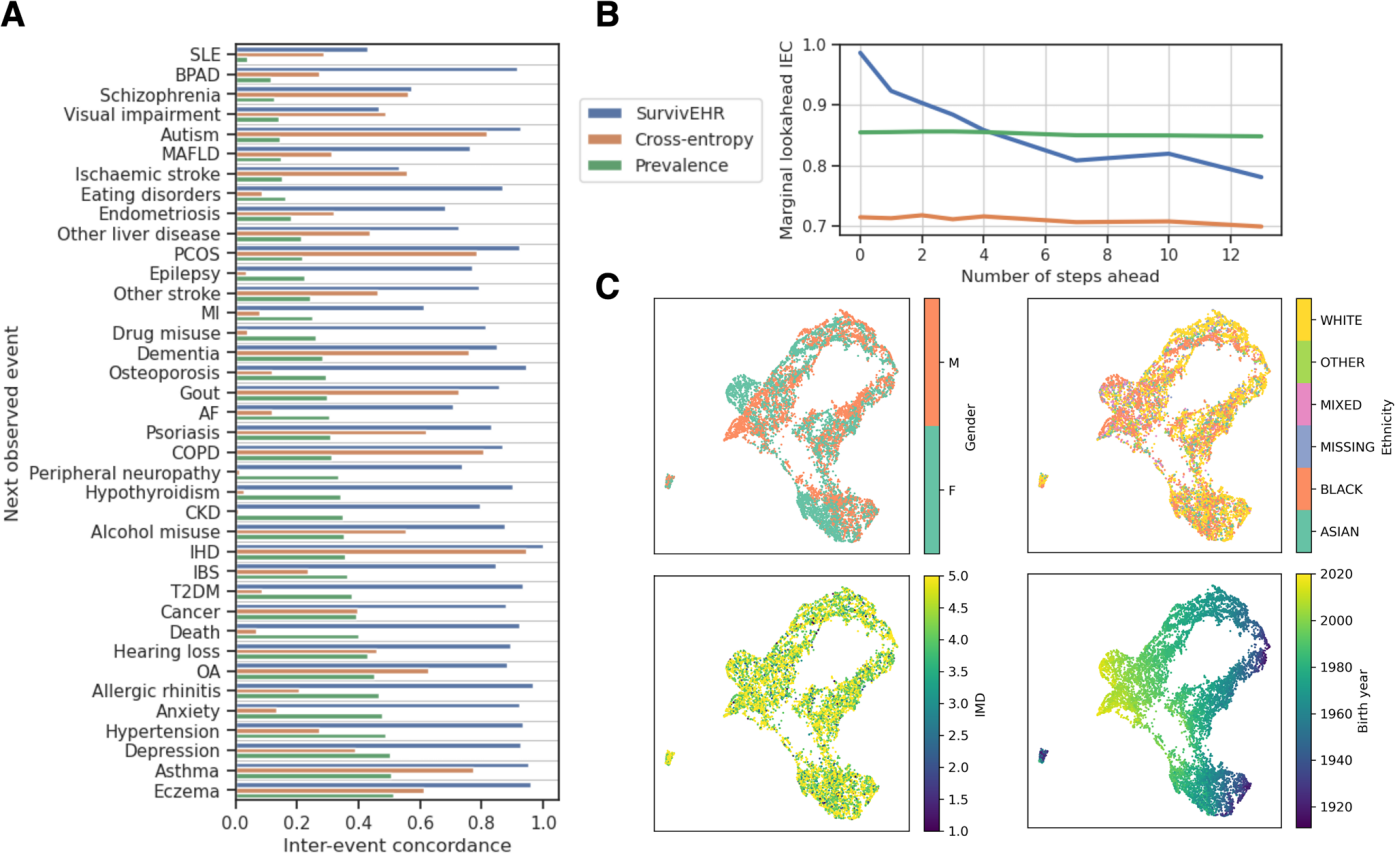
Evaluation of SurvivEHR on the pre-training dataset. **A** Each row gives the inter-event concordance score, which quantifies the model’s ability to correctly rank next-event risk within patient timelines. **B** Marginal inter-event concordance for predicting events increasingly into the future. As we predict further into the future, we observe a reduction in the performance of foundation models. **C** UMAP representations of high-dimensional latent encodings that preserve local neighbourhood structure, labelled by sex, ethnicity, index of multiple deprivation (IMD), and age.

SurvivEHR demonstrated strong self-supervised predictive performance compared to the baselines (Fig. 4A, Supplementary Fig. S2). We obtain a marginal IEC of 0.994, meaning that on average, the true next event was among the top (1–0.994) × 263 ≈ 1.6 predicted events, demonstrating extremely strong overall predictive performance. Alternatively, following the baseline strategy of diagnosing by prevalence led to a score of 0.864, meaning that, on average, the true next event was among the top 36 most prevalent events. Finally, using the typical multi-label classification, cross-entropy training objective led to an IEC score of 0.699, meaning that on average, the true next event was among the top 80 predicted, illustrating that discretisation of a time-to-event problem can be detrimental.

### Multi-step forecasting

During pre-training, SurvivEHR was tasked with predicting the next event. However, particularly when used for prognostic forecasting, we may be interested in the prediction of arbitrary future outcomes, rather than the immediate subsequent event. We tested the capacity of SurvivEHR to perform multi-step predictive forecasting in the pre-training test cohort. In Figure 4B, we used our marginal IEC score to evaluate how well SurvivEHR was able to stratify competing risks over an increasing time horizon, comparing this to the previous baseline approaches. Given a patient’s history, we used SurvivEHR to predict the first, second, third and beyond future events. We emphasise that recursive multi-step generation is used solely as a qualitative assessment of generative plausibility and learned trajectory structure, rather than as a calibrated estimator of time-to-event risk. Accordingly, no survival discrimination or calibration metrics are reported for recursive steps beyond the immediate next event. We observed that a clear degradation in performance occurs, such that after four steps, it is no longer able to outperform the prevalence-based baseline. This indicates a distributional shift between the pre-training (next step) and evaluation (two-four steps forward) tasks. This is unsurprising since SurvivEHR is built upon a modified GPT structure and shares conceptually parallel challenges seen in LLMs and other related clinical foundation models, in doing multi-step forecasting or long-horizon reasoning, which have prompted recent developments in remedies such as chain-of-thought reasoning.

### Interpreting SurvivEHR properties and behaviours

SurvivEHR compresses each patient history into a 384-dimensional latent embedding vector, which encodes all the information held in that record. We explored, by projecting these 384-vectors into two-dimensional visualisations using UMAP^42^, what information was contained within these embeddings. Figure 4C and Supplementary Figs. S3 and S4 show four two-dimensional projections of the latent embeddings for a random subset of the pre-training cohort, each coloured by different sociodemographic factors. These projections did not show clear large-scale stratification with respect to gender, ethnicity, or the IMD. However, we emphasise that such low-dimensional visualisations are descriptive and do not provide sufficient evidence to assess the presence or absence of bias or fairness-related effects. Formal evaluation of subgroup performance and fairness would require dedicated quantitative analyses beyond the scope of this study. Accordingly, these visualisations should be interpreted as exploratory summaries of embedding structure rather than definitive assessments of representation quality across demographic groups.

We further explored if SurvivEHR is learning predictive patterns from the primary care records by identifying the clinical significance of the next event forecasts made by SurvivEHR. We use a recursive strategy to forecast a short sequence of future clinical trajectories and analyse the top marginal occurrence rates and the pairwise co-occurrence matrix that captures how often events arise together. Using the pre-training cohort, we present SurvivEHR with each held-out patient’s complete history and ask it to predict the next *three* clinical events (we only predict the next three events since previous analysis shows breakdown after four steps). For every pair of events, we compute the one-step transition probability from one event to the next event. This exploratory approach was designed to qualitatively assess whether SurvivEHR recovers well-established clinical relationships.

**Fig. 5:**
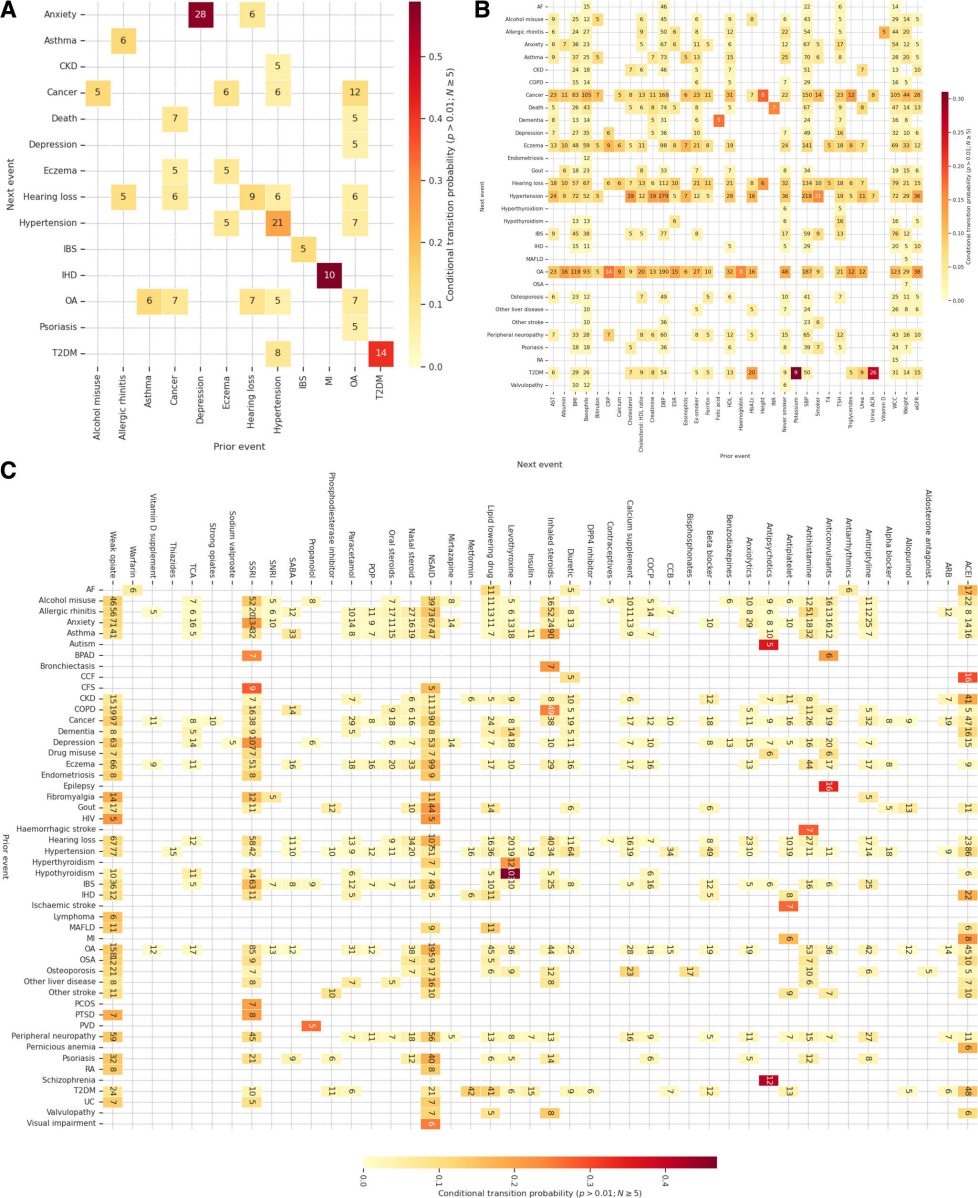
Forecasting known clinical associations. Given unseen patient histories from the pre-training cohort, we predict the next 3 events a patient may experience. From these, we plot the next event occurrences matrices for **A** a new diagnosis following a previous diagnosis, **B** a diagnosis follows an investigation, and **C** medication follows a diagnosis. Colour scales indicate the SurvivEHR-derived conditional next event occurrence probabilities, while numbers indicate the observed occurrences during forecast generation. Next event occurrences with fewer than 15 occurrences and frequency below 0.05 are removed for clarity.

Figure 5A and Supplementary Fig. S5 show counts and conditional next event occurrences from SurvivEHR for an illustrative subset of diagnoses. Here, there are strong conditional next event occurrences for well-known clinical relationships such as Depression→Anxiety, which is frequently observed in chronic mental health progression^43^, Type 2 Diabetes (T2DM)→Osteoarthritis (OA) (obesity and systemic inflammation)^44^ and Osteoarthritis (OA)→Myocardial infarction (MI), which is a well-characterised epidemiological association^45^. These relationships mirror established multimorbidity pathways, suggesting that SurvivEHR can uncover latent structures useful for clinical decision support.

When we examined what SurvivEHR would predict as possible diagnoses following investigations (Fig. 5B, Supplementary Fig. S7), we observed that SurvivEHR predicts hypertension as a next event, often after systolic and diastolic blood pressure (SBP/DBP) measurements, as expected. However, preceding cholesterol, creatinine, and eGFR measurements were also associated with hypertension diagnoses, and these are routinely measured when there is clinical suspicion of hypertension. Weight, body mass index (BMI), and smoking status were also associated with preceding events. We also found the expected transition that type 2 diabetes mellitus (T2DM) diagnoses follow blood glucose (HbA1c) measurements, which was also recovered, which is consistent with clinical practice in the UK^46^.

Finally, we examined which medications were forecast by SurvivEHR following a diagnosis (Fig. 5C, Supplementary Fig. S6). In the case of T2DM, these were metformin (a standard first-line treatment) and lipid-lowering and ACE inhibitors, which are routinely prescribed to address the increased risk of cardiovascular and kidney disease in T2DM patients^46^. Schizophrenia was followed by antipsychotics. Epilepsy by anticonvulsants. Osteoporosis was followed by prescriptions of bisphosphonates—a gold standard, first-line treatment—and calcium supplements, which are supportive medications for the condition^47^. However, as an indication that SurvivEHR predictions are not *causal*, we see that osteoporosis was also associated with subsequent non-steroidal anti-inflammatory drugs (NSAIDs). NSAIDs are not indicated for osteoporosis itself but could be prescribed to manage associated (pain) symptoms or comorbid conditions. Similarly, visual impairment itself is not an indication for prescribing SSRIs (selective serotonin reuptake inhibitors). However, SurvivEHR’s forecasts have likely learned that SSRIs may be prescribed in people with visual impairment if they have associated mental health conditions, such as depression or anxiety, which are common in individuals coping with vision loss.

**Fig. 6:**
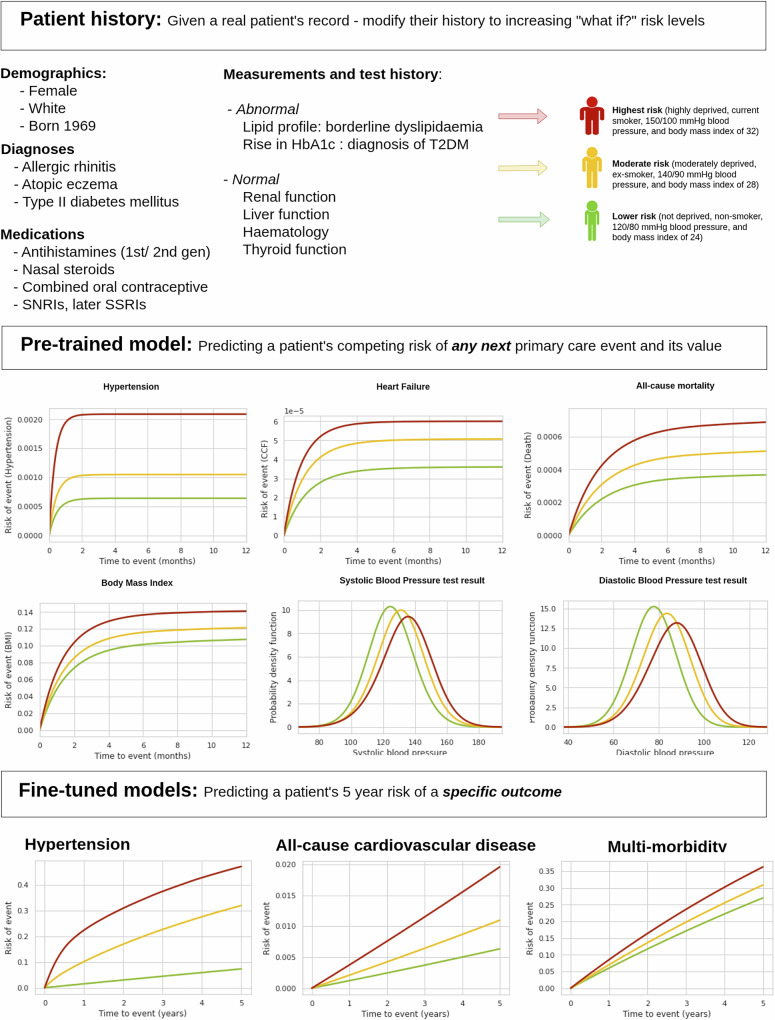
Illustrating SurvivEHR predictive behaviour. We constructed a hypothetical patient and modified their body weight, smoking and blood pressure histories towards three (low, medium and high) levels of increasing risk. We show the corresponding cumulative incidence functions (CIF) for BMI measurement, all-cause mortality, type 2 diabetes mellitus, heart failure, and hypertension from both the pre-trained and fine-tuned models, showing the changes in profile across the three risk levels.

Similarly, we also considered how risk profiles for individual patients change by varying different aspects of their history. To demonstrate this, we curate a hypothetical female patient, born in 1963 and of Asian ethnicity. We model their history upon a real patient from the pre-training held-out cohort who did not experience any morbidities. Given this history of blood pressure, weight, blood test, smoking status monitoring, and previous anti-inflammatory drug medications, we can then modify this to transcribe our patient into three potential risk categories by varying their risk factors. In each case, we mask any recorded weight values to ensure the record remains self-consistent. We define three categories: (i) low risk: a non-smoker, 120/80 mmHg blood pressure, and BMI of 24, (ii) moderate risk: an ex-smoker, 140/90 mmHg blood pressure, and BMI of 28 and (iii) high risk: a current smoker, 150/100 mmHg blood pressure, and BMI of 32. Given these histories, we can then forecast what their future risk for different events would be. Figure 6 shows the CIF predicted by SurvivEHR, demonstrating that high-risk individuals with obesity are more likely to have BMI measurements taken again than the other risk groups. While the risk of mortality, T2DM, heart failure and hypertension is also significantly increased, as would be expected.

### Clinical risk prediction and fine-tuning performance

We next evaluated SurvivEHR on clinically motivated risk prediction tasks designed to reflect common prognostic questions in primary care following a diagnosis of type 2 diabetes (Fig. 8A). Specifically, we assessed 5-year risk, a time horizon commonly used in primary care to support medium-term prevention, monitoring, and service planning. The first task involved predicting incident hypertension, a frequent and clinically actionable comorbidity in people with type 2 diabetes that represents a classic single-outcome risk prediction scenario. The second task considered CVD as a competing-risk outcome, defined by the mutually exclusive onset of ischaemic heart disease or stroke (see ‘Methods’), reflecting the need to model multiple adverse but interrelated outcomes that are central to MLTC management.

For benchmarking predictive performance, we constructed time-to-event risk prediction models using Penalised Cox models as well as ensemble and DL approaches (random survival forests (RSFs)^48^, DeepHit^12^, DeepSurv^49^ and DeSurv^13^) using a cohort of 572,096 patients. We note that Cox, RSF, DeSurv, DeepSurv and DeepHit use cross-sectional (most-recent) inputs and not full patient histories; they provide an important reference for contextualising performance improvements observed with SurvivEHR. The alternative models (Table 1) that use longitudinal input do not provide competing risk capability. Because of the substantial computational demands, we limit comparisons with pre-trained foundation models to the regional analysis described later.

We compared these models to predictions from two fine-tuned versions of SurvivEHR. The first was a scratch fine-tuned (SFT) version, which uses the SurvivEHR architecture but was not pre-trained. The second version used full fine-tuning (FFT), which was first pre-trained before being fine-tuned for the 5-year prediction task. This allows us to examine whether any performance improvement is due simply to having a complex architecture (SFT) or if the pre-training adds information (FFT). We also tested the zero-shot (ZS) performance of SurvivEHR on this task by evaluating how well the pre-trained model can perform this task without any task-specific training.

We report three standard metrics, the time-dependent concordance measures how well a model is able to distinguish risk between patients who experience an event at different times; the Integrated Brier Score quantifies how well the model can accurately predict the probability of an event occurring over a period; whilst the Integrated Negative Binomial Log-Likelihood measures how well the model prediction matches the actual occurrence of the event (Further results stratified by demographic are given in Supplementary Table S8).

**Fig. 7:**
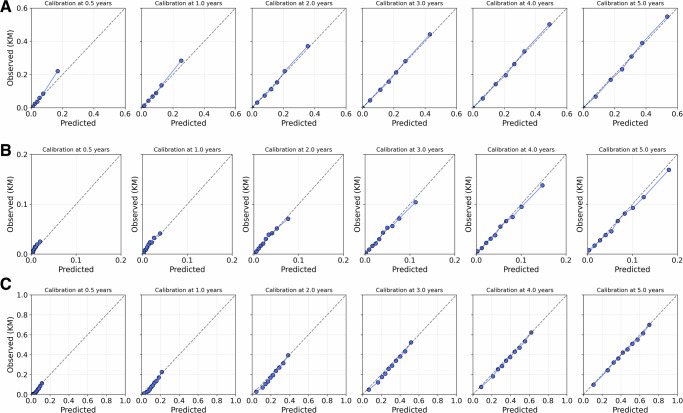
Calibration of SurvivEHR predictions across clinical outcomes and time horizons. Calibration plots comparing predicted and observed event risk at 0.5, 1, 2, 3, 4, and 5 years for three downstream prognostic tasks. Row **A** shows 5-year risk prediction for incident hypertension, while row **B** for cardiovascular disease (CVD) under a competing-risk formulation, and row **C** shows multimorbidity progression, defined as the onset of additional long-term conditions. Each point represents a risk decile, with the dashed line indicating perfect calibration. Across outcomes and time horizons, SurvivEHR demonstrates good agreement between predicted and observed risks, supporting its use for calibrated risk stratification following fine-tuning.

**Fig. 8:**
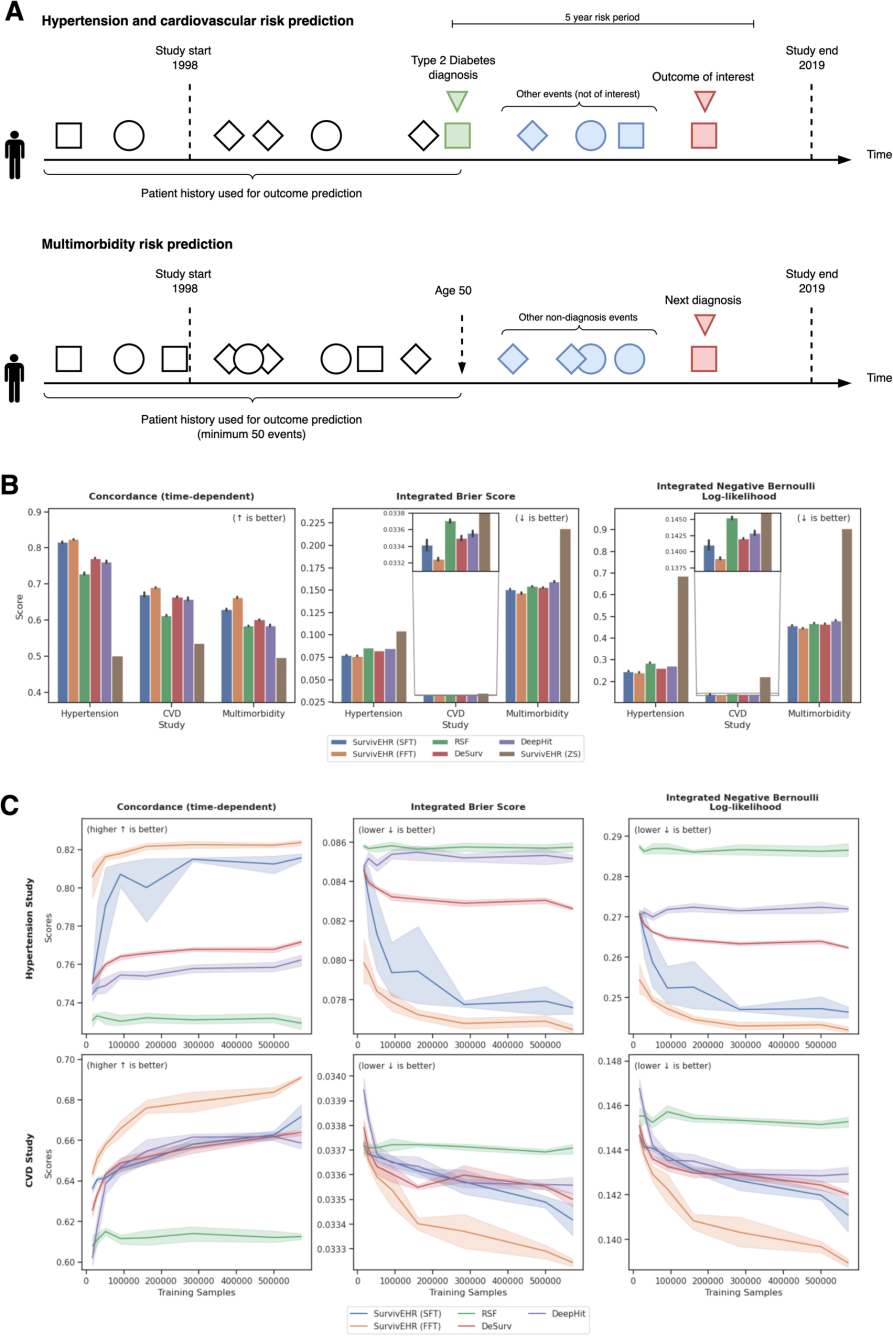
Fine-tuning prediction performance evaluation. **A** Fine-tuned models for retrospective cohort studies are trained and evaluated on contexts up to and including the index date. Subsequent events are excluded until either (i) an outcome is observed or (ii) the last observation within the study period, which is then treated as a censored target. **B** Survival performance metrics for each cohort. **C** Ablation study of increasing cohort study population size. Figure created using draw.io.

Figure 8B (Table 2) shows that SurvivEHR-FFT achieves superior predictive performance to the other benchmarks on both tasks across all metrics for both prediction tasks. This demonstrates that pre-training provided additional prior information and that improved predictive performance did not arise purely due to increased model complexity and use of longitudinal input over RSF, DeSurv, DeepHit and SurvivEHR-SFT. SurvivEHR predictions were also well-calibrated for both predictive tasks (Fig. 7A, B).

**Table 2:** Clinical risk prediction model performance using all regions

Case study	Model	Type	*C*_td_ (↑)	IBS (↓)	INBLL (↓)
5-year hypertension risk		Zero-shot	0.561	0.105	0.602
	SurvivEHR	SFT	0.816 ± 0.003	0.0776 ± 0.0005	0.246 ± 0.002
		FFT	**0.824** ± **0.002**	**0.0765** ± **0.0003**	**0.242** ± **0.001**
	Random Forest	-	0.729 ± 0.005	0.0857 ± 0.0004	0.286 ± 0.002
	Penalised CoxPH		0.769 ± 0.000	0.0832 ± 0.0000	0.265 ± 0.000
	DeSurv	-	0.772 ± 0.002	0.0826 ± 0.0001	0.262 ± 0.000
	DeepHit	-	0.762 ± 0.004	0.0852 ± 0.0003	0.272 ± 0.001
	DeepSurv	-	0.771 ± 0.001	0.0827 ± 0.0001	0.263 ± 0.0002
5-year cardiovascular disease risk		Zero-shot	0.555	0.0354	0.222
	SurvivEHR	SFT	0.648 ± 0.004	0.0336 ± 0.0000	0.143 ± 0.001
		FFT	**0.691** ± **0.001**	**0.0332** ± **0.0000**	**0.139** ± **0.000**
	Random Forest	-	0.613 ± 0.002	0.0337 ± 0.0000	0.145 ± 0.0004
	Penalised CoxPH	-	0.665 ± 0.000	0.0334 ± 0.0000	0.142 ± 0.000
	DeSurv	-	0.664 ± 0.002	0.0335 ± 0.0000	0.142 ± 0.0002
	DeepHit	-	0.659 ± 0.006	0.0336 ± 0.0001	0.143 ± 0.0005
	DeepSurv	-	0.667 ± 0.0006	0.0335 ± 0.00002	0.142 ± 0.0001
5-year multi-morbidity risk	SurvivEHR	SFT	0.629 ± 0.003	0.151 ± 0.000	0.459 ± 0.001
		FFT	**0.663** ± **0.002**	**0.147** ± **0.001**	**0.446** ± **0.003**
	Random Forest	-	0.584 ± 0.001	0.154 ± 0.0002	0.469 ± 0.0005
	Penalised CoxPH	-	0.601 ± 0.001	0.154 ± 0.0002	0.468 ± 0.0005
	DeSurv	-	0.601 ± 0.001	0.153 ± 0.0003	0.466 ± 0.0007
	DeepHit	-	0.561 ± 0.006	0.157 ± 0.0004	0.477 ± 0.0009
	DeepSurv	-	0.600 ± 0.001	0.153 ± 0.0005	0.467 ± 0.0011

Zero-shot indicates the evaluation of a pre-trained model, without any cohort-specific training. Scratch Fine Tuning (SFT) indicates the risk prediction model was trained from scratch. Full Fine Tuning (FFT) indicates that we initialised training from the pre-trained model. The survival metrics (*C*_td_, IBS and INBLL) evaluate the model’s ability to predict the final outcome, and we report the average and 95% confidence interval over five random seeds. Bold indicates the best performing method.

These improvements were more evident if the patient cohort size was reduced from 572,096 patients to something substantially smaller. Figure 8C shows that when the cohort size is reduced, fine-tuning from a pre-trained model helps to maintain predictive performance, and there are substantial benefits if the fine-tuning cohort size is less than 100,000 over the alternative models considered. Further, using a pre-trained model significantly improves model stability, with models trained from scratch leading to higher performance variability.

We next considered a more challenging risk prediction task focused on multimorbidity progression: predicting the development of additional long-term conditions from age 50 in patients with heterogeneous pre-existing multimorbid health profiles. In this setting, the outcome was defined as the onset of one or more new long-term conditions from a predefined list of 74 conditions (see ‘Methods’), reflecting clinically meaningful disease accumulation rather than single-disease incidence. Age 50 was chosen as a clinically relevant index point at which multimorbidity commonly emerges and preventive interventions may still alter longer-term trajectories. As before, SurvivEHR achieved superior predictive performance across all metrics for this complex multimorbidity prediction task compared with the other baseline models (Figs. 7C and 8B and Table 2).

These results demonstrate that fine-tuning from a single pre-trained foundation model is able to enhance performance for different and variable predictive tasks. However, we note that zero-shot predictive performance is not attained by SurvivEHR, which is to be expected since its pre-training objective is next-event prediction and not prediction over longer time horizons. This will be discussed in more detail later.

### Improving predictive performance with limited cohorts

We next tested the ability of SurvivEHR to support clinical prediction model development in the context of a limited cohort. Prediction models are often constructed from data arising from a single regional area, health system, or population subgroup. This is often as a result of data access barriers, which limit the ability to utilise more representative training sets and to perform external validation. Unfortunately, this can put models at an elevated risk of failing to generalise to other populations or the original scope^50^.

Motivated by known regional differences in demographics (such as deprivation and ethnicity, see Fig. 3), we first tested the generalisability of SurvivEHR by creating two regional cohort groups: those from the North East of England (total sample size 765,091) and those from London (total sample size 6,678,166). We first pre-trained SurvivEHR on patients from the North East, attaining an IEC score of 0.9916. This demonstrates a strong performance in comparison to our earlier baselines, despite the limited number of pre-training samples available. To assess the ability of SurvivEHR to generalise to unseen pre-training populations, we then evaluate on the held-out data from the London population, attaining an IEC score of 0.9831. A reduction of 0.0085 in IEC represents a <1% decline in predictive ranking accuracy, indicating strong transferability.

We next repeated our previous benchmarking on this reduced cohort, introducing a comparison to the BEHRT^36^ due to the smaller cohort size. BEHRT uses Masked Language Modelling on the longitudinal input as the pre-training objective. However, as this method does not provide time-to-event or competing risk capability, during fine-tuning, we replaced the classification head with the same DeSurv head used by SurvivEHR. We trained SurvivEHR-SFT, BEHRT, RSF, DeSurv and DeepHit each from scratch on the North-East region data and compared to SurvivEHR-FFT which was fine-tuned on the North-East data. Models trained or fine-tuned using data exclusively from one region and evaluated on a held-out test set from the same or an alternate region.

Table 3 shows that fine-tuning from the pre-trained model allows SurvivEHR-FFT to achieve improved predictive performance relative to the other models trained only on the North East set across all tasks and metrics, demonstrating the benefits of using a generic pre-trained model to augment a more limited training set.

**Table 3:** Clinical risk prediction model benchmark performance in the North East

Case study	Method	Type	*C*_td_ (↑)	IBS (*↓*)	INBLL (↓)
5-year hypertension risk	SurvivEHR	SFT	0.524	0.0849	0.309
		FFT	**0.825**	**0.0726**	**0.236**
	BEHRT	SFT	0.557	0.0856	0.313
		FFT	0.668	0.0817	0.284
	Random Forest	-	0.734	0.0787	0.271
	Penalised CoxPH	-	0.776	0.0761	0.252
	DeSurv	-	0.746	0.0788	0.259
	DeepHit	-	0.735	0.0830	0.271
	DeepSurv	-	0.753	0.0789	0.259
5-year cardiovascular disease risk	SurvivEHR	SFT	0.620	0.0404	0.168
		FFT	0.637	**0.0400**	**0.166**
	BEHRT	SFT	0.517	0.0408	0.171
		FFT	0.573	0.0408	0.171
	Random Forest	-	0.605	0.0405	0.169
	Penalised CoxPH	-	**0.666**	0.0415	0.168
	DeSurv	-	0.637	0.0401	0.166
	DeepHit	-	0.626	0.0404	0.167
	DeepSurv	-	0.617	0.0409	0.170
5-year multi-morbidity risk	SurvivEHR	SFT	0.614	0.163	0.486
		FFT	**0.640**	**0.157**	**0.474**
	BEHRT	SFT	0.556	0.171	0.509
		FFT	0.573	0.167	0.500
	Random Forest	-	0.578	0.165	0.495
	Penalised CoxPH	-	0.595	0.163	0.491
	DeSurv	-	0.593	0.164	0.492
	DeepHit	-	0.587	0.173	0.513
	DeepSurv	-	0.603	0.161	0.485

Change in performance when Scratch Fine Tuning (SFT, in which the model is trained from scratch) versus Full Fine Tuning (FFT, in which we initialised training from the pre-trained model). The survival metrics (*C*_td_, IBS and INBLL) evaluate the model’s ability to predict the final outcome. Each model is fine-tuned and evaluated on the cohort from the North East of England. Bold indicates the best performing method.

### Fine-tuning improves predictive performance in the context of distributional shift

Having established that SurvivEHR performs well in small cohorts, we next examined whether this robustness extends to populations with differing demographic structures. Specifically, we assess how well SurvivEHR—pre-trained on nationwide data—transfers knowledge when fine-tuned on data from one region before being subsequently applied to another region, reflecting a realistic deployment scenario. We adopted the same hypertension, CVD and multimorbidity prediction tasks as before, and for each, we trained SurvivEHR on London and North East data, respectively. We constructed versions based on scratch training (SFT) purely on the regional dataset and also fine-tuned models initialised from the nationwide pre-trained model (FFT).

Table 4 shows results from the various combinations of training and test scenarios. For example, when SurvivEHR-SFT was trained on the London region and tested on London and North East-based test sets, the concordance index (*C*_td_) was 0.813 and 0.822, respectively. However, this improved to 0.835 and 0.846 when using fine-tuning from the pre-trained model SurvivEHR-FFT. In general, we see pre-training leads to an improvement in predictive performance, even when evaluating on a different subpopulation with a different demographic distribution. Further, in many cases a FFT model trained on a region different to the one it was evaluated on performed better than one trained from scratch in the target population.

**Table 4:** Clinical risk prediction model performance between regions

Case study	Training	Evaluation	*C*_td_ (↑)	IBS (↓)	INBLL (*↓*)
				(SFT→FFT)	
5-year hypertension risk	London	London	0.813 → 0.835	0.0748 → 0.0720	0.241 → 0.231
		North East	0.822 → 0.846	0.0709 → 0.0675	0.230 → 0.218
	North East	North East	0.524 → 0.825	0.0849 → 0.0726	0.309 → 0.236
		London	0.519 → 0.811	0.0897 → 0.0772	0.321 → 0.249
5-year cardiovascular disease risk	London	London	0.651 → 0.686	0.0297 → 0.0296	0.130 → 0.127
		North East	0.638 → 0.666	0.0403 → 0.0400	0.167 → 0.163
	North East	North East	0.620 → 0.650	0.0404 → 0.0403	0.167 → 0.166
		London	0.643 → 0.635	0.0299 → 0.0298	0.133 → 0.132
5-year multi-morbidity risk	London	London	0.617 → 0.652	0.158 → 0.153	0.476 → 0.462
		North East	0.615 → 0.640	0.162 → 0.157	0.484 → 0.473
	North East	North East	0.614 → 0.640	0.163 → 0.157	0.486 → 0.474
		London	0.609 → 0.645	0.158 → 0.155	0.476 → 0.468

another. Shown is the change in performance when training with scratch fine-tuning (SFT) on the training population, versus full fine-tuning (FFT) on the training population from a model pre-trained using all nationwide data. The survival metrics (*C*_td_, IBS and INBLL) evaluate the model’s ability to predict the final outcome. Each model is fine-tuned on a cohort from either London or the North East of England and then evaluated on both cohorts. We show that pre-training improves performance even when evaluating out of the fine-tuning population.

## Discussion

We have described a foundation model (SurvivEHR) that supports the development of clinical risk prediction models for patients with MLTCs. The foundation model leverages primary care EHRs that offer rich longitudinal trajectories that span decades of patient care, encompassing the full spectrum of health states from wellness monitoring through chronic disease management to end-of-life care. We exploit EHR data for a substantive proportion of the UK population to create a decoder-only transformer architecture that is pre-trained using a competing risk, time to next event objective. By improving risk stratification across multiple chronic conditions, SurvivEHR could support the development of novel predictive models that could help in the prioritisation of proactive interventions in primary care, especially in data-scarce settings.

In UK primary care, patients with MLTCs are managed through continuous risk assessment and prioritisation rather than single-disease pathways. By jointly modelling competing risks across multiple outcomes, SurvivEHR captures patient trajectories in a way that reflects real-world clinical decision-making. This capability may support proactive identification of high-risk patients, prioritisation of follow-up and monitoring, and downstream analysis of care patterns in complex populations.

Our experiments indicate that SurvivEHR can recapitulate known clinical associations and risk factor relationships, validating the fundamental premise that primary care EHRs contain learnable patient trajectories. Critically, these associations emerged without explicit programming or clinical rules, suggesting that the model architecture successfully learned the underlying epidemiological structure embedded within primary care practice patterns. These insights, captured in the embedding properties of SurvivEHR, can be exploited using fine-tuning to create more powerful clinical risk prediction models that utilise the insights gleaned from the extensive population records seen and captured by SurvivEHR.

Due to sensitivity and privacy issues, access to individual-level patient medical records, like those provided by CPRD, is typically highly restricted and granted under limited conditions of use after extensive review and approval of an intended use plan. Pre-trained foundation models like SurvivEHR can provide an alternative approach for data sharing and model development by providing embedding functions pre-captured from individual-level data without the need to access or share the individual records themselves. As such, while our specific instance of SurvivEHR is trained only on a UK population and its most prevalent MLTCs, we envisage that similar models could be trained on other datasets, particularly in different health systems, which would account for variations in practice and clinical care guidelines. However, one issue of note is that while our experiments highlighted that multi-step forecasting capability in SurvivEHR was limited, we cannot provide full guarantees that the generative capability of SurvivEHR could not recapitulate real individual records in CPRD (e.g. those individuals with few coded events, particularly involving rare conditions), and this currently places a limitation on model sharing. This is a general issue to be considered for all generative EHR models.

Our findings suggest that SurvivEHR captures realistic and clinically meaningful dependencies within primary care data. However, this analysis was intended to provide illustrative rather than exhaustive evidence of learned associations. A systematic comparison across all possible event pairs was not attempted because such an evaluation would require (i) defining an extensive set of ground-truth associations across thousands of diagnostic, laboratory, and medication codes; (ii) accounting for population- and practice-specific variations in data recording and clinical pathways; and (iii) managing the substantial computational and interpretive burden of testing the tens of millions of possible event transitions contained within the dataset. We again note that this experiment should not be considered causal prediction^51^ since SurvivEHR only learns associative and not causal relationships.

Accordingly, the examples presented here should be interpreted as qualitative demonstrations of plausibility rather than a quantitative validation of clinical correctness. Future work could extend this analysis using curated ontologies (e.g. ULMS^52^) or epidemiological (e.g. Global Burden of Disease Study^53^) benchmarks to enable more systematic validation of learned clinical dependencies.

We demonstrate that the zero-shot long-term predictive performance of SurvivEHR remains limited due to its next-event pre-training objective. Future work could incorporate multi-step objectives or explicit long-range prediction targets to address these limitations. In primary care, risk prediction is often used to support preventative decision-making over extended periods, and the choice of prediction horizon can influence clinical interpretation. However, horizon-specific optimisation is not the focus of this work; rather, our aim is to demonstrate SurvivEHR as a general foundation model for constructing time-to-event predictors across a range of outcomes and data regimes. The development and validation of guideline-level long-horizon risk scores would require outcome-specific design choices and dedicated evaluation and are therefore beyond the scope of this methodological study.

We further note that SurvivEHR was constructed only from coded medical data and did not use potentially free-text data that exists in primary care records. Free text data is omitted from the research data provided by CPRD, and the limitations and potential biases of this omission have been previously studied^54^. Future model developments could integrate free text and other data modalities, though it should be noted that there is often limited availability of integrated population health data for research and a lack of computational and data infrastructure within clinical settings for the implementation of predictive analytics that might make use of such data.

The performance gains observed with SurvivEHR arise from the combination of several interacting factors, including longitudinal sequence conditioning, large-scale self-supervised pre-training, and joint competing-risk modelling. While our benchmarking experiments demonstrate that pre-training materially improves downstream performance relative to training from scratch using the same architecture, we do not claim a full causal decomposition of these effects. A complete factorial ablation across architecture, input representation, pre-training objective, and competing-risk formulation (such as Sumo-NET^55^ and Neural Fine-Gray^56^) would be computationally prohibitive at the scale of data required for stable estimation and is beyond the scope of this work.

Similarly, recent GPT-based foundation models for EHRs (e.g. ETHOS, CEHR-XGPT) model longitudinal patient trajectories using autoregressive sequence modelling (see ‘Methods’ for a more detailed discussion). While these approaches can capture complex temporal dependencies and generate plausible future event sequences, they do not explicitly model censoring or competing risks in the survival analysis sense. As a result, they do not directly yield equivalent time-to-event risk estimates for specific outcomes over defined horizons. In contrast, SurvivEHR is trained with a competing-risk time-to-event objective, enabling direct estimation of CIFs across multiple outcomes. We emphasise that differences in objective function—particularly the explicit treatment of censoring and time-to-event distributions—may lead to qualitatively different behaviours compared to autoregressive models, even when trained on similar longitudinal data. Therefore, a systematic empirical comparison between autoregressive generative models and censoring-aware survival foundation models represents an important direction for future work.

Instead, our evaluation strategy is designed to reflect realistic modelling choices faced in applied clinical prediction settings, where access to large-scale longitudinal data and pre-training resources is uneven. By comparing SurvivEHR to both classical survival models and neural baselines trained on equivalent cross-sectional representations, we aim to contextualise its performance gains while acknowledging that no single benchmark can isolate all contributing factors. Importantly, the consistent improvements observed under reduced cohort sizes and regional distributional shift suggest that pre-training and longitudinal conditioning confer robustness advantages that are clinically meaningful even when attribution cannot be fully disentangled.

Finally, as an associative machine learning model, SurvivEHR learns predictive functions from observed patterns in the data. When trained on CPRD, these patterns reflect not only underlying disease processes, but also healthcare practice norms shaped by local policies, incentives, coding conventions, and care pathways within the UK primary care system. As a result, this instantiation of SurvivEHR is naturally tailored to the UK primary care, and direct transportability of learned predictive patterns to other health systems may be limited by differences in operational practices and data recording. Future work could investigate approaches to disentangle aetiological signals from healthcare practice-specific effects, or to adapt and fine-tune the model using locally representative data, in order to improve generalisation across healthcare jurisdictions.

In summary, SurvivEHR adds a distinctive methodological contribution to a growing body of work in foundation models for EHR by providing one of the first decoder-only foundation models to learn full competing risk, time-to-event distributions.

## Methods

### Clinical Practice Research Datalink

We used the CPRD^20^, which contains longitudinal primary care data from a network of over 2000 general practices in the UK that use EMIS Web® patient record software. Around 1 in 10 GP practices, with 10 million currently registered patients and another 30 million historical patient records, contribute data to CPRD. CPRD is broadly representative of the population by age, sex, and ethnicity. It has been extensively validated and is considered as the most comprehensive longitudinal primary care database, with several large-scale epidemiological reports adding to its credibility. Access to CPRD Aurum data was approved as part of the ‘OPTIMising therapies, disease trajectories, and AI-assisted clinical management for patients Living with complex multimorbidity’ (OPTIMAL) study^35^ with approval from the CPRD Independent Scientific Advisory Committee (protocol number: 21_000683). Our study is a retrospective observational analysis of routinely collected primary care EHRs. No human participants were prospectively assigned to any health-related interventions, and no interventions were delivered as part of this research.

### Data pre-processing

CPRD data was extracted using DExtER^33^. Clinical observations, diagnoses and treatments are recorded as Read Version 2, SNOMED-CT, and EMIS Web® clinical codes. Prevalent cases for all 74 long-term conditions were identified using disease-specific clinical codelists. Codelists for 81 classes of medications associated with the management of the 74 conditions were identified from prescription data recorded as Prodcodes from the Dictionary of Medicines and Devices (DM+D) codes, which are a subset of the SNOMED-CT terminology. We used 108 types of measurement and test (investigation) results, with values, reflecting routine blood tests such as haemoglobin, total cholesterol or serum creatinine levels, and clinical measurements such as blood pressure readings that are also routinely recorded in CPRD (Supplementary Table S1). Codelists can be obtained from GitHub (https://github.com/aditya02acharya/optimal_data). This gives a total of 263 (74+81+108) competing risks where every diagnosis, medication and investigation is considered an event.

In addition, we also record birth year, male-female gender, regional area, and the IMD for each individual. IMD is an area-level composite measure of socioeconomic deprivation in England, derived from national indicators across seven domains: income, employment, education and skills, health and disability, crime, barriers to housing and services, and living environment. In CPRD, IMD is assigned based on patient postcode at registration and is used as a static baseline covariate reflecting contextual socioeconomic conditions rather than individual-level deprivation.

The list of conditions, investigations and medications to be included was previously determined through an iterative process of discussion amongst a group of clinical experts, including GPs, public health consultants and geriatricians, with further guidance from our patient advisory group. The starting point for these discussions was the findings of a seminal Delphi study that identified 59 key conditions that should be included in research for patients with MLTCs^34^ and UK NIHR-funded AI for Multimorbidity research consortia, which included OPTIMAL^4^. Our consortium extended this list to 74 conditions based on additional patient and expert advisory input. Relevant laboratory tests and medications were selected according to their established clinical relevance to these conditions. As our CPRD data access is governed by pre-registered and ethically approved protocols, experiments involving random or frequency-ranked task selection are not currently feasible.

Our systematic process of developing clinical codelists and drug codelists using the DExtER code builder tool has been described in detail elsewhere^35^. We considered patients from England with at least 12 months of acceptable data recording prior to the index date (1 January 2005). Acceptable data was determined using the ‘acceptable patient flag’ data quality measure provided by CPRD: (consistent recording of events including date of birth, practice registration date and transfer out date, and valid age and sex). There were 26.5 million patients across 1478 practices meeting our inclusion criteria.

Pre-processing was performed using FastEHR, a custom Python toolkit that implements a high-throughput extract-transform-load machine learning data pipeline for EHR data extracts. It ingests raw data, builds an indexed SQLite store, and then uses the Polars library to stream large tables through memory-efficient transformations and filters. The pipeline handles: tokenisation; data cleaning, such as outlier removal and de-duplication; and allows custom dataset and dataloader curation for pre-training and fine-tuning. Users can specify flexible inclusion, exclusion, indexing and pre-processing criteria suitable for both self-supervised pre-training and supervised fine-tuning, whilst controlling for general practice- and dataset-level data leakage. In our workflow, FastEHR was developed as the data pre-processing backbone preceding model training with the SurvivEHR foundation model and is made available alongside the SurvivEHR package.

Following pre-processing with FastEHR, we were left with 51,003,640 diagnoses, 3,970,984,804 medications, and 3,533,426,831 investigations, of which 3,364,437,065 included the accompanying investigation result (Supplementary Table S2). Of these records, we employed a 90-5-5 site-level training, validation and test random split, dividing patients by general practices in England to avoid data leakage across practices. This left 23,613,894 patients from 1330 practices in the training cohort, 1,426,714 patients from 74 practices in the validation cohort, and 1,508,320 patients from 74 practices in the test cohort. Statistics of the data extracted through DExtER and bounds used for internal scaling and outlier removal by FastEHR can be found in the Supplementary Information (Supplementary Tables S3–S6). Continuous value measurements were zero-centred.

### Existing work

DL-based prediction models vary by architecture but broadly fall under three categories: encoder-only, encoder-decoder and decoder-only. Encoder-only models (e.g., Med-BERT^57^, BEHRT^36^) use masked language modelling or contrastive learning to learn contextual representations and are well-suited for classification and embedding tasks. Encoder-decoder models (e.g. TransformEHR^39^) support sequence-to-sequence tasks, are similarly trained, and are effective for summarisation, question answering, and multi-task learning via prompting. Whilst decoder-only models (e.g. ETHOS^58^, Foresight^41^) use autoregressive training for generation, and scale well for large modelling and generative applications. A summary of existing DL-based clinical risk prediction models is given in Table 1. For brevity, we include only those clinical foundation models built for structured clinical codes rather than other modalities, including free text and images.

In this work, we are interested in decoder-only approaches for next-event prediction. Many next-event prediction architectures have been used as base structures for EHR models. Each of these approaches typically adopts a two-stage training approach. Pre-training is first performed on large-scale longitudinal EHRs using self-supervised objectives to learn general-purpose representations of clinical features. This is then followed by fine-tuning on downstream clinical tasks such as diagnosis prediction, treatment recommendation, or risk stratification. However, by using architectures originally designed for language modelling, many EHR-based prediction models are limited to classifying the next most likely diagnosis or outcome at the next patient visit, and are either unable to provide a prediction for when this event will occur, or are only able to provide a point prediction.

Consequently, without explicit modelling of event timing, these models cannot properly account for censoring and important temporal risk information is lost, which can limit the clinical utility. For example, they cannot be used to directly answer questions such as ‘What is this patient’s risk of heart failure in the next X months?’ for any arbitrary future time period. While previous models often include time or age-based embeddings to model the temporal order and distancing between events, this is not the same as learning time-to-event distribution models, which can automatically account for irregular and variable follow-up durations.

This limitation was first overcome with MOTOR^18^, a self-supervised, time-to-event (TTE) foundation model that leverages a Transformer encoder with a piecewise exponential survival head to learn temporal risk representations from large-scale EHR data. MOTOR is pre-trained across thousands of pseudo-tasks that predict the distribution of time until specific medical events, optimising a likelihood based on a piecewise exponential hazard formulation. This architecture enables the model to capture complex, nonlinear dependencies between longitudinal clinical events while efficiently scaling to millions of patients. Once pre-trained, MOTOR can be adapted to downstream prognostic tasks via linear probing or FFT, providing improved time-dependent concordance and calibration compared to traditional Cox, DeepSurv, and DeepHit models.

Other TTE models have also recently been developed, such as CEHR-XGPT^59^, which models time distributions using Gamma distributions and Delphi-2M^15^, which uses exponential distributions, but these also require specialised time or no-event tokens to be incorporated. Interestingly, it is important to highlight that the handling of temporal information can be influenced implicitly by the type of data available to model developers. For example, in CEHR-XGPT^59^, intervals exceeding 1080 days are denoted by a long-term token which have low frequency of occurrence in the training data from a tertiary care centre (Columbia University Irving Medical Center-New York Presbyterian Hospital). This would not be applicable to the UK primary care data we utilise since intervals exceeding 1080 days are relatively common.

Time-gap tokenisation can be an effective strategy for incorporating temporal information in longitudinal models, particularly in settings with relatively short and bounded inter-event intervals. CLIMBR^60^ employs coarse time bins (e.g. 24 h, 1–7 days, 8–30 days, >31 days) tailored to hospital-based outcomes, while ETHOS^58^ represents elapsed time using a fixed set of interval tokens ranging from minutes to 6 months, with longer gaps approximated via repeated tokens.

Primary care records exhibit highly irregular inter-event intervals that frequently span many years or decades (Fig. 2D). In this setting, time-gap tokenisation would require either the introduction of specialised long-term tokens or long sequences of repeated short-interval tokens, both of which introduce discretisation artefacts and substantially increase sequence length.

While ETHOS and similar models can generate future trajectories via autoregressive sampling, they do not explicitly account for censoring in the survival-analysis sense; sequences are truncated at the last observed event, and timing information is learned implicitly through autoregressive prediction rather than through a censoring-aware time-to-event likelihood.

### Generative pre-trained transformers

The objective of SurvivEHR was to predict the time to the next recorded primary care event given a stream of previous primary care encounters and events (the patient history). This problem is highly related to generative modelling and is a form of self-supervised learning since histories can be retrospectively partitioned to set up supervised prediction tasks. Generative models built on this underlying task have become extremely popular in recent years due to their strong zero-shot generalisation^61^, apparent emergent capabilities^62,63^ and scaling laws^64^. GPT models learn representations of sequences and, regardless of the form of these sequences, require each element to be vectorised. This is typically achieved through a process known as tokenisation and is then usually followed by a projection of each token. An attention mechanism then captures dependencies between tokens, allowing the model to weigh the importance of each token relative to others, regardless of their position in the sequence. This process allows GPT models to capture contextual relationships and generate coherent outputs by predicting the next token based on prior ones, effectively learning patterns and relationships within the sequence over time.

For each patient, we have a temporal sequence of primary care events that occur at irregular intervals. Each element of this sequence is broken down into a tuple of three items: a categorical event index, corresponding to a unique combination of medical codes; any attached numerical value, such as an investigation result; and finally, the event time, in days to/since birth. The modelling task is to predict the next event and the associated time-to-event distribution, given the baseline covariates and the sequence of previous records. For instance, this may be predicting the risk of the next event being a diagnosis of substance abuse, and the corresponding time-to-event. In this example, no corresponding target value exists. This is visually depicted in Fig. 1B.

### Neural-based time-to-event distributions and competing risks

Crucially, SurvivEHR extends this generative formulation to a competing risks framework, recognising that at any point in a patient’s timeline, multiple mutually exclusive clinical events may occur next. Rather than modelling a single event type in isolation, SurvivEHR jointly learns cause-specific CIFs for each possible next event, allowing it to estimate both which outcome is most likely to occur and when. This is achieved by coupling the transformer’s latent state representation with a neural survival head^13^ that outputs CIFs across all event types, ensuring that the model captures the dependencies and mutual exclusivity among diverse clinical outcomes. As a consequence, SurvivEHR makes no parametric assumptions about TTE distributions, unlike those used in CEHR-XGPT^59^ or Delphi-2M^15^ and does not require special time tokens.

SurvivEHR also goes further than MOTOR^18^, which formulates time-to-event prediction as a multi-task, single-risk problem, where each outcome (for example, heart attack, stroke, or diagnosis code) is treated as an independent survival task with its own prediction head and piecewise exponential likelihood. During pre-training, MOTOR computes a separate hazard estimate for each task and time interval, using task-specific embeddings to project shared patient representations into multiple condition-specific hazard spaces. While this enables large-scale parallelisation across thousands of survival tasks, it assumes conditional independence between outcomes and handles competing events, such as death, by treating them as censoring events.

SurvivEHR, by contrast, integrates these outcome processes into a single competing-risk generative model. Instead of maintaining separate prediction heads for each condition, it employs a unified survival head that jointly parameterises cause-specific CIFs across the full event vocabulary. This design allows SurvivEHR to model shared temporal dependencies and interactions between outcomes directly, without assuming independence or discarding censored data. The result is a calibrated, probabilistic representation of both the timing and the type of the next event, aligning its predictions with the complex, interdependent trajectories observed in longitudinal primary care data.

### Electronic health record embeddings

We describe the procedure used to vectorise individual EHR events. Each event consists of: (i) a categorical event type (defined over a vocabulary of medical codes), (ii) an optional associated measurement value, and (iii) continuous time since birth. In addition to longitudinal events, EHRs also contain static patient-level information (e.g., sex or baseline demographics) that remains constant across the record. The objective is to map each event to a learned numerical 384-dimensional representation suitable for downstream modelling.

For each patient, we include a representation of their baseline covariates that remain fixed throughout their EHR. These include sex, ethnicity, year of birth, and postcode-derived IMD. As patient records from different general practices cannot be linked within CPRD, each patient is associated with a single registration. Consequently, derived IMD scores remain constant over the recorded history.

To accommodate missingness explicitly, categorical variables are encoded using one-hot representations (including IMD, despite its ordinal structure), while year of birth is treated as a continuous covariate. The resulting encoded features are concatenated into a single vector **x** and mapped into the model’s latent space via a learned linear projection,

1 $${{\bf{e}}}^{{\rm{static}}}={W}^{{\rm{static}}}{\bf{x}}\in {{\mathbb{R}}}^{384}.$$

Each EHR event occurring over the course of a patient’s lifetime is represented using a *split* embedding. Specifically, at the *c*th position in a patient’s timeline, we seek to construct a numerical representation of the recorded event category *p*_*c*_ together with any associated measurement value *v*_*c*_. To achieve this, we adopt a split architecture composed of two embedding networks: one that encodes the categorical event identity, and a second that models the associated value when (when present).

2 $${{\bf{e}}}_{c}^{{\rm{event}}}={W}^{(1)}\left({W}^{{\rm{event}}}[{p}_{c}]\right)\in {{\mathbb{R}}}^{384},$$

3 $${{\bf{e}}}_{c}^{{\rm{value}}}={W}^{(2)}\left({v}_{c}{W}^{{\rm{value}}}[{p}_{c}]\right)\in {{\mathbb{R}}}^{384}.$$

Here, *W*^event^[*p*_*c*_] and *W*^value^[*p*_*c*_] denote the rows of the respective embedding matrices indexed by the event token. The value contribution is therefore event-specific and scaled by the (zero-centred) observed measurement *v*_*c*_, allowing deviations of values from the population average to refine the base event representation.

The categorical event identity embedding determines which clinical process has occurred and, therefore, must be identifiable regardless of the scale of value or whether it was observed. In contrast, the associated value (when present) provides additional quantitative information conditional on the event type. Many event categories (e.g., diagnoses or prescriptions) do not carry numeric values, and thus a representation based solely on value channels would fail to distinguish between distinct non-valued events. Explicit encoding of event identity, therefore, acts as an indicator mechanism, ensuring that each event type is uniquely identifiable within the model.

By default, the Transformer architecture is agnostic to the order in which events occur. As a consequence, it is typical to encode the positional information in each record embedding, encapsulated as the number of days from birth to when the *c*th event occurred, *t*_*c*_.

There are many strategies for this^65,66^, each accounting for irregular times at which events can occur. Our experiments with learnt positional embeddings showed no benefit for the increased computational cost, motivating us to use the original positional encoding of^65^:

4 $${{\bf{e}}}_{({t}_{c},2d)}^{{\rm{time}}}=\sin \left({t}_{c}/1000{0}^{2d/{d}_{{\rm{hid}}}}\right),$$

5 $${{\bf{e}}}_{({t}_{c},2d+1)}^{{\rm{time}}}=\cos \left({t}_{c}/1000{0}^{2d/{d}_{{\rm{hid}}}}\right).$$

Each of these embeddings is additively combined to obtain the record embedding. Although the static embedding variables do not change over time, we additionally include their embedding with each event to provide context consistently across the entire sequence.

6 $${{\bf{E}}}_{c}={{\bf{e}}}^{{\rm{static}}}+\frac{1}{2}\left({{\bf{e}}}_{c}^{{\rm{event}}}+{{\bf{e}}}_{c}^{{\rm{value}}}\right)+{{\bf{e}}}_{c}^{{\rm{time}}}.$$

Putting these event embeddings up to event **E**_*c*_ into the Transformer provides us with a representation of a patient’s medical history, denoted by **T**_*c*_ (Supplementary Fig. S1).

### Survival and value pre-training prediction

Autoregressive Language Modelling predicts the next word in a sequence based solely on the preceding context encoded as **T**_*i*_, following the causal structure of natural language generation. Here, our goal is to replace the typical black-box network, which predicts next token logits, with a new approach which instead predicts the full time-to-event survival distribution. This can then be used within the generative language modelling framework to infer a patient’s future sequence of health records.

Given a sequence of health records **r**_*c*_ = (*p*_*c*_, *v*_*c*_), occurring at irregular times *t*_*c*_, this translates to predicting the next event **r**_*c*+1_, and the time-to-event Δ*t*_*c*+1_ = *t*_*c*+1_ − *t*_*c*_ by providing accurate personalised cumulative distribution functions of the survival time. That is, given the history of a test subject, be able to provide an accurate estimate of the distribution over the survival time for each next event that may occur, and any value associated with this event.

Given a medical history encoded through **T**_*c*_, survival analysis aims to predict the varying risk of a future event *p*_*c*+1_ (distinguishing event type), over the time-to-event Δ*t*_*c*+1_. A single risk approach assumes each of the possible next events can be considered in isolation, whilst a competing risk approach assumes only one of the possible next events can occur in lieu of the others.

We consider the case of competing risks. In the decoder-only setting, we instead have *k*_*c*+1_ ∈ {1, …, 263}. By default, the self-supervised learning is well-suited for the competing risk case, as each subsequent event may be only one of the considered events (under the assumption that event times follow a Poisson process).

Further, this means no null event $\varnothing$ is typically needed during pre-training. Though semantically, we can consider vocabulary truncation (in which events of low frequency are excluded) to form this set. In the case of pre-training for competing risks, the CIF associated with each event type is defined as:

7 $${F}^{\left(p\right)}(t| {{\bf{T}}}_{c})=P(T\le t,p| {{\bf{T}}}_{c}).$$

This is related to the CIF for the overall survival time (until any next event) by

8 $$F(t| {{\bf{T}}}_{c})=\mathop{\sum }\limits_{p}{F}^{\left(p\right)}(t| {{\bf{T}}}_{c})\,\mathrm{where}\,p=1,\ldots ,263.$$

In each case, the Transformer’s hidden states, **T**_*c*_, are used as input to a deep survival model to predict survival outcomes. For this, we choose to use DeSurv^13^, a flexible neural network-based approach for modelling survival distributions in continuous time. However, alternatives such as Cox-based survival models (e.g. PyCox^67^) or alternative neural approaches (e.g. SumoNet^55^, Neural Fine-Gray^56^) could also be used. However, due to the computational demands of experiments with EHR data of this scale, it is beyond the scope of the current work to perform a comparative assessment of the relative benefits of these potential alternatives.

### Generation and value prediction

Our survival heads predict a cumulative density for each separate event over a fixed time grid. Sampling the time-to-event within each is achieved through a two-stage approach.

First, we sample which event will occur next with a probability proportional to the area under the cumulative density over the 5-year period.

Secondly, we sample the time-to-event using inverse transform sampling.

For each valued-event (such as those events *p* for which a value *v* may exist), we define a probabilistic regression layer predicting a Gaussian mean and standard deviation, conditional upon the hidden representation **T**_*i*_. The resultant log-likelihood is then combined with the loss additively. This approach comes with a limitation that predicted values share the same distribution across all future times. Consequently, we will not observe a pharmacological onset time. This simplification is similarly made in existing state-of-the-art approaches, and is left as future work.

### Pre-training objective specification

We model competing-risk CIFs ${F}_{\theta }^{(p)}(t| x)$ using a derivative-based continuous-time formulation in the spirit of DeSurv^13^. Specifically, each CIF is obtained by integrating an ODE whose derivative is parameterised by a neural network sub-module.

For each cause *p* ∈ {1, …, *K*}, define a latent ODE state ${u}_{\theta }^{(p)}(t| x)$ via

9 $$\frac{{\rm{d}}}{{\rm{d}}t}\,{u}_{\theta }^{(p)}(t| x)={g}_{\theta }^{(p)}\left(t,\,x\right),\,\,{u}_{\theta }^{(p)}(0| x)=0,$$

where ${g}_{\theta }^{(p)}(t,x)$ is a neural network ensuring a non-negative derivative for monotonicity. The cause-specific CIF is then

10 $${F}_{\theta }^{(p)}(t| x)=\sigma \,\left({u}_{\theta }^{(p)}(t| x)\right),$$

with *σ*(⋅) a smooth squashing function such as $\tanh$ or a softplus (ensuring ${F}_{\theta }^{(p)}(t| x)\in [0,1]$). The corresponding instantaneous density is

11 $${f}_{\theta }^{(p)}(t| x)=\frac{{\rm{d}}}{{\rm{d}}t}\,{F}_{\theta }^{(p)}(t| x)={\sigma }^{{\prime} }\,\left({u}_{\theta }^{(p)}(t| x)\right)\,{g}_{\theta }^{(p)}\left(t,x\right).$$

All neural network components ${g}_{\theta }^{(p)}$ and *σ* share parameters *θ*. This construction guarantees valid, continuous-time CIFs without restrictive parametric assumptions.

Given the above, we now provide a full specification of the pre-training objective function. Let ${T}_{c}={T}_{\theta }({{\mathcal{H}}}_{c})$ denote the transformer latent state parameterised by *θ*, obtained from the patient history ${{\mathcal{H}}}_{c}$ up to event *c*. The model is trained autoregressively to predict the next event type *k*_*c*+1_ ∈ {1, …, *K*} (with *K* = 263 competing risks), the time-to-next-event Δ*t*_*c*+1_, and (when applicable) an associated continuous value *v*_*c*+1_. With this DeSurv parameterisation, the competing-risk survival negative log-likelihood becomes

12 $${{\mathcal{L}}}_{{\rm{surv}}}(\theta )=-\mathop{\sum }\limits_{n=1}^{N}\mathop{\sum }\limits_{c}\log {f}_{\theta }^{({k}_{c+1}^{(n)})}\,\left(\Delta {t}_{c+1}^{(n)}| {T}_{c}^{(n)}\right),$$

where

13 $${f}_{\theta }^{(p)}(t| {T}_{c})={\sigma }^{{\prime} }\,\left({u}_{\theta }^{(p)}(t| {T}_{c})\right)\,{g}_{\theta }^{(p)}\left(t,{T}_{c}\right),$$

and total CIF

14 $${F}_{\theta }(t| {T}_{c})=\mathop{\sum }\limits_{p=1}^{K}{F}_{\theta }^{(p)}(t| {T}_{c}).$$

For valued events, the Gaussian value prediction head remains:

15 $${v}_{c+1} \sim {\mathcal{N}}\left({\mu }_{\theta }({T}_{c}),{\sigma }_{\theta }^{2}({T}_{c})\right),$$

with

16 $${{\mathcal{L}}}_{\mathrm{val}}(\theta )=-\mathop{\sum }\limits_{n=1}^{N}\mathop{\sum }\limits_{c\in {{\mathcal{V}}}^{(n)}}\log \,{\mathcal{N}}\,\left({v}_{c+1}^{(n)}| {\mu }_{\theta }\left({T}_{c}^{(n)}\right),{\sigma }_{\theta }^{2}\left({T}_{c}^{(n)}\right)\right).$$

The combined loss is

17 $${{\mathcal{L}}}_{\mathrm{total}}(\theta )={{\mathcal{L}}}_{\mathrm{surv}}(\theta )+{\rm{\lambda }}\,{{\mathcal{L}}}_{\mathrm{val}}(\theta ),$$

where *λ* controls the trade-off between survival and value prediction and was set to *λ* = 0.1, and parameters *θ* are learned by

18 $$\widehat{\theta }=\arg \mathop{\min }\limits_{\theta }{{\mathcal{L}}}_{{\rm{total}}}(\theta ).$$

Model hyperparameters used for training are given in Supplementary Table S7.

### Pre-training evaluation metrics

Survival metrics evaluate the performance of models predicting time-to-event outcomes, whilst accounting for unique challenges like censoring. Key metrics include the concordance index (c-index), which assesses the model’s ability to correctly rank relative risks between patients, the Integrated Brier Score (IBS), measuring time-dependent prediction accuracy, and the Negative Bernoulli Log-Likelihood (NBLL), which quantifies the likelihood of observed survival times given model predictions. These metrics enable rigorous comparisons and validation of survival models, ensuring robust and reliable predictions. In part, they are able to achieve this by accounting for censored samples, which represent incomplete but valuable information about survival times. Improper handling of censored samples leads to a poor reflection of the true predictive performance of survival models in real-world scenarios where complete data is rare.

Concordance metrics, for example, quantify the consistency between the relative predicted risk between patients, compared to the observed risk. If patients *i* and *j* both experienced an event, the concordance is higher if the model correctly predicts which patient experiences the event first. This *intra*-event metric is individually calculated for each event across concordant pairs of patients. Many estimates of this metric for survival analysis exist, with the simplest being Harrell’s c-index, which counts the proportion of concordant pairs

19 $$\frac{{\sum }_{i}{\sum }_{j}I({y}_{i} < {y}_{j})I({\delta }_{i}=1)I({R}_{i} > {R}_{j})}{{\sum }_{i}{\sum }_{j}I({y}_{i} < {y}_{j})I\left({\delta }_{i}=1\right)},$$

where *i* and *j* index patient pairs, *y*_*i*_ is the minimum of the survival time *t*_*i*_ and censoring time ${u}_{i},\,\delta =I\left({t}_{i}\le {u}_{i}\right)$, and *R*_*i*_ is an estimated risk score under some model^68^.

However, event sparsity in EHRs makes within-event pair construction computationally impractical. For example, rare events such as Addison’s disease occurred at a frequency of 6691 diagnoses in 7.6 billion events, making within-batch computation practically infeasible. Instead, we propose a new metric based upon the discrimination approach of Harrell’s c-index, but instead considering between-event discrimination. This leads to a metric similar to those found in recommender systems, such as the Mean Reciprocal Rank.

We employ a new metric to assess a pre-trained model’s capacity to order survival risk between events in the self-supervised setting.

We are interested in developing an *inter*-event metric that is capable of determining consistency in the relative predicted risk of all possible next events, compared to the observed true next event.

In these settings, rather than quantify the ability to capture relative risks between pairs of patients, we quantify the accuracy of the pre-trained model in correctly ranking the risks of the next true event, across all possible next events. In doing this, we obtain a discrimination score for each individual event category, quantifying how accurately the model is able to rank the relative risk.

We now update the above index notation: given all cases where an event $i\in \left\{1,\ldots ,263\right\}$ occurred next, we derive a score quantifying how well the risk of this event was ranked across all 263 possible predicted next events, *j*

20 $${{\mathcal{P}}}_{i}\approx \frac{1}{{N}_{{\rm{test}}}}\mathop{\sum }\limits_{n=1}^{{N}_{{\rm{test}}}}\left(\frac{{\sum }_{c=2}^{{C}_{n}}I\left({p}_{c}^{(n)}=i\right)\left(\frac{1}{263}{\sum }_{j=1}^{263}I\left({\widehat{R}}_{c,i}^{(n)}\ge {\widehat{R}}_{c,j}^{(n)}\right)\right)}{{\sum }_{c=2}^{{C}_{n}}I\left({p}_{c}^{(n)}=i\right)}\right),$$

where ${p}_{c}^{(n)}$ is the index of the event experienced by patient *n* after experiencing *c* prior events. Similarly, ${\widehat{R}}_{c,i}^{(n)}$ is the predicted estimated risk score for the next event *i*, for patient *n*, after observing the *c* prior events. For the risk score, we use the restricted mean survival time (RMST) calculated over a 5-year period. This is marginalised over both the number of patients *N*_test_, and each new event the patient experienced *C*_*n*_. In practice, we obtain an unbiased approximation of this by sub-sampling.

Through this approach, we can quantify model alignment with the training objective, identify patterns that the model predicts confidently and highlight potential biases or weaknesses in its learned representations. This approach is particularly useful for understanding event-level prediction fidelity and for comparing the model’s performance across various pre-training datasets and tasks. Such metrics based on Harrell’s c-index^69^ are known to be a function of the censoring distribution^70,71^, inflating the scores of more prevalent events. This is similarly true for our proposed metric, and consequently, we compare our model to a baseline score, which is obtained by following a prevalence-only prognostic approach, in which the risk score ${\widehat{R}}_{i}$ is equal to the total number of events *i* in the population.

The *IEC* scores for each medication and investigation are shown in Fig. 4A. These are calculated across every new event observed within each patient’s history for a subset of the unseen held-out patients. In each of these, some events may be missing due to sub-sampling.

### Multi-step forecasting

We can use the Inter-event Concordance metric to evaluate how performance degrades as we look further ahead by updating (20).

Retaining the notation for event index $i\in \left\{1,\ldots ,263\right\}$, we derive a score quantifying how well the risk of an event *t*-steps in the future is ranked across all 263 possible future events, *j*:

21 $${{\mathcal{P}}}_{i,t}\approx \frac{1}{{N}_{{\rm{test}}}}\mathop{\sum }\limits_{n=1}^{{N}_{{\rm{test}}}}\left(\frac{I\left({p}_{c+t}^{(n)}=i\right)\left(\frac{1}{263}{\sum }_{j=1}^{263}I\left({\widehat{R}}_{c,i}^{(n)}\ge {\widehat{R}}_{c,j}^{(n)}\right)\right)}{I\left({p}_{c+t}^{(n)}=i\right)}\right).$$

Here, *c* is chosen to be the index of the event *t* steps prior to each patient’s last observation, such that *c* + *t* is the number of records in the timeline of patient *n*. We do not calculate the score over the entire patient trajectory, but include the medical history up to *t*-steps before their final observation, and test the ability to predict the final observed event. Consequently, ${p}_{c+t}^{(n)}$ is the final event category experienced by patient *n*. We then evaluate how well the model was able to predict the risk of this event multiple steps in the future, despite being trained to predict ${p}_{c}^{(n)}$.

### Fine-tuning experiments

In our fine-tuning experiments, we consider the downstream task of predicting the risk of a future outcome from a patient history up to an index date. Throughout all fine-tuning examples, we increase the context window to 512, prepend any previous diagnoses which may otherwise be lost from the context window, and remove repeated events. This ensured that all health conditions that could have lifelong implications were included for prediction. All benchmark examples are given full patient histories, and therefore are not limited by context length.

We considered two approaches to fine-tuning. The first we call supervised fine-tuning from scratch (SFT), in which the supervised model is trained from randomly initialised parameters. While in the second, we perform supervised FFT from the pre-trained model. This allowed us to distinguish between learning due to the model architecture and that which occurs through transfer learning. Additionally, we benchmark against a number of alternative methods to predict the 5-year risk using Survival Random Forests^48^, DeepHit^12^, and DeSurv^13^, Cox and DeepSurv^49^. For each, we take a cross-section of the full patient history, including baseline covariates, all diagnoses, medications and investigations. This is composed of a concatenation of the baseline covariates and a binary vector, where cases where an event occurred before an index date are marked as 1, and 0 otherwise. Note that we could not include approaches (such as BEHRT) which process longitudinal input data but only provide single-risk, binary classification output for comparison.

We fine-tune on two different cohorts. For the first cohort, we consider patients with T2DM and indexing at this diagnosis. After applying our inclusion criteria, we are left with 572,096 patients, with an additional 35,758 reserved for validation and 33,280 for testing. Within this cohort, we consider both single- and competing-risk tasks of predicting the 5-year risk of hypertension and CVD, respectively. We took CVD to be the competing-risk of ischaemic heart disease (including myocardial infarction) and stroke (including ischaemic stroke, haemorrhage, and from unspecified causes). For our second cohort, we considered patients with pre-existing multimorbidity, indexing at age 50 and using a random sample of 20,000 patients for training. We predict the 5-year risk of acquiring any new health condition (any of the 71 long-term conditions that the individual does not already have).

A full outline of the inclusion criteria for each study is provided in the Supplementary Information.

## Supplementary information

Supplementary Information

## Data Availability

Data for this project were made available by CPRD under study reference ID 21_000683 (https://www.cprd.com/approved-studies/optimising-therapies-and-disease-trajectories-patients-living-complex). Raw data from the study are not publicly available. Data for the study were obtained under licence from CPRD; pseudonymised patient data are available from CPRD subject to Research Data Governance approval; see https://www.cprd.com/how-access-cprd-data for more information. Codelists for CPRD data extraction can be found at http://github.com/THINKINGGroup/phenotypes.

## Code availability

The full source code used to pre-process, train, evaluate, and reproduce our experiments is available at http://github.com/cwlgadd/FastEHR and http://github.com/cwlgadd/SurvivEHR under an open-source licence. As the proposed SurvivEHR model uses generative AI, we are not able to distribute the trained model weights to avoid compromising patient privacy and contravening data-sharing agreements, as we cannot guarantee that exact copies of real records could be reproduced by the model. We provide detailed instructions to allow others to retrain the model from scratch on appropriately licensed data. Access to an embeddings-only pre-trained model without decoding ability may be made available upon request to the corresponding authors.
