# Supplementary material for: SurvivEHR: a competing risks, time-to-event foundation model for multiple long-term conditions from primary care electronic health records
Source: NPJ Digit Med. 2026 May 9;9:546. doi: 10.1038/s41746-026-02709-z (PMC13376826; doi:10.1038/s41746-026-02709-z)
Supplement: Supplementary file 1 — Supplementary Information [file 41746_2026_2709_MOESM1_ESM.pdf]

Supplementary Material:
SurvivEHR: a competing risks, time-to-event foundation model for multiple long-term conditions from primary care electronic health records

Charles Gadd[1], Krishna Gokhale[2], Aditya Acharya[2], Jennifer Cooper[2], Francesca Crowe[2], Leah Fitzsimmons[2], Thomas Jackson[2], Krishnarajah Nirantharakumar[2,3], Christopher Yau[1,4], and The OPTIMAL collaborative[*]

[1]Nuffield Department for Women's & Reproductive Health, University of Oxford, Oxford, UK.
[2]University of Birmingham, Birmingham, UK.
[3]Kings College London, London, UK.
[4]Health Data Research UK, London, UK.

Contents

A Data processing **3**
A.1 DExtER . . . 3
A.1.1 Code list . . . 3
A.1.2 Summary statistics . . . 3
A.2 FastEHR . . . 3
A.2.1 Tokenisation . . . 3
A.2.2 Outlier filtering and scaling . . . 4
A.2.3 Missing data . . . 4

B Pre-training performance evaluation **5**
B.1 Representation Learning and demographic bias . . . 5
B.2 SurvivEHR captures known clinical associations . . . 5

List of Tables

S1 SurvivEHR event vocabulary . . . 6
S2 Event counts . . . 11
S3 Summary statistics of diagnoses extracted by DExtER . . . 11
S4 Summary statistics of medications extracted by DExtER . . . 13
S5 Summary statistics of investigations extracted by DExtER . . . 14
S6 Pre-processing bounds . . . 17
S7 Model hyperparameters used during pre-training and fine-tuning. . . . 20
S8 Clinical risk prediction model performance stratified by demographics . . . 21

List of Figures

S1 Decoder architecture . . . 22
S2 Inter-event concordance . . . 23
S3 Learnt representations . . . 24
S4 Learnt representations with baseline covariates masked . . . 25
S5 Forecasting in pre-training cohort: diagnosis to diagnosis . . . 26

S6 Forecasting in pre-training cohort: diagnosis to medication 27
S7 Forecasting in pre-training cohort: investigation to diagnosis 28
S8 Forecasting in pre-training cohort: investigation to diagnosis 28

A Data processing

A.1 DExtER

CPRD Aurum data is extracted according to a pre-specified study protocol using DExtER [1]. Under this protocol, DExtER extracts 74, 81 and 108 collections of diagnosis, medication and investigation codes, respectively. These are outlined in Supplementary Table S1, where we additionally show the corresponding labels used for analysis and plotting. The total number of records extracted by DExtER is shown in Supplementary Table S2.

A.1.1 Code list

The list of conditions, medications and investigation to be included was determined through an iterative process of discussion amongst a group of clinical experts including general practitioners, public health consultants and geriatricians, with further guidance from our patient advisory group. The starting point for these discussions was the findings of a seminal Delphi study that identified 59 key conditions that should be included in research for patients with multiple long term conditions (MLTCs). Our systematic process of developing clinical codelists and drug codelists using the DExtER codebuilder tool has been described in detail elsewhere. Codelists can be found at `http://github.com/THINKINGGroup/phenotypes`.

A.1.2 Summary statistics

Summary statistics for the data extracted using DExtER, before any pre-processing is performed, are given in Supplementary Tables S3–S5. Count refers to the total number of examples extracted from CPRD. For investigations, for which we also have numerical observations, we also report the mean, minimum, and maximum statistics across observations. Additionally, we report the number of occurrences when an event occurred but the associated value was unavailable.

These statistics are obtained before any pre-processing steps are applied and demonstrate the irregular structure of the raw data, including both sparsity and wide variation in numerical recording. As a consequence of this we performed extensive pre-processing, including outlier removal outlined in Section A.2.2, and chose to mask poorly recorded values relating to smoking status.

A.2 FastEHR

We developed FastEHR, an open-source Python package, to construct high-throughput machine learning datasets for structured electronic health records (`http://github.com/cwlgadd/FastEHR/`). Upon extraction of raw data by DExtER, we built an indexed, single-file database using SQLite which can be used to collate patient information and collect meta information used for pre-processing and reporting. This is then used to build flexible datasets that allow for different training objectives, study inclusions, cohort curation, and different outcomes.

FastEHR supports scalable preprocessing via Polars, patient- and practice-level inclusion criteria, configurable cohort splits, and different tokenization strategies. Additionally, FastEHR outputs model-ready datasets in Parquet format alongside metadata for reproducibility, and provides data loaders compatible with PyTorch, making it an efficient backbone for large-scale EHR model development.

A.2.1 Tokenisation

We employed whole-word tokenisation, where each token corresponds to an event category composed of the collection of medical codes for diagnoses, medications, or investigations extracted using DExtER. The full list of these event categories, their corresponding token index, and the short format names used for analysis and plotting are shown in Supplementary Table S1.

The first two tokens are reserved for internal padding (“PAD") and an unknown (“UNK") token. Whilst unused in our experiments, the unknown token is designed to encapsulate any out-of-vocabulary event

categories, flagging them so the model can still process sequences containing unknown events, even though it lacks a dedicated embedding for that item.

A.2.2 Outlier filtering and scaling

Feasible lower (F_L) and upper (F_U) outlier limits were chosen by clinical experts. For those values where clinician-derived limits were not available, we instead derived empirical bounds, defined by $F_L = Q_1 - 1.5 * IQR$ and $F_U = Q_3 + 1.5 * IQR$, where Q_1 and Q_3 are lower and upper quantiles, and IQR is the inter-quantile range. Quantiles were approximated by sub-sampling with t-Digest [2]. The limits used are given in Supplementary Table S6.

Investigations whose values lay beyond these limits had their values removed during pre-processing and were subsequently treated as missing. In these cases, the token was still retained and consequently the model remains aware that the investigation occurred, but not what the investigation result was.

Internal scaling was performed on the remaining recorded values using min–max scaling on these limits. Motivated by our choice of embedding architecture, we chose to centre our standardisation at zero.

A.2.3 Missing data

Missingness is a common characteristic of electronic health records, particularly for investigation codes where numerical values may be absent or improperly recorded. We distinguish between missing values in the input (predictor) and target (self-supervised or supervised label) contexts.

For missing input investigation results we apply a simple imputation strategy. After applying the outlier removal and scaling procedures described above, missing values are set to zero, which corresponds to imputing the midpoint of the interquartile range, $\frac{Q_1+Q_3}{2}$.

For missing target values, we retain the remainder of the patient record but exclude the missing values from contributing to the loss function during training. This allows the model to learn from partially observed outcomes.

We are unable to account for cases where an event may have occurred but the medical code was not recorded in the patient's records. This is a consequence of their absence being unobservable and thus indistinguishable from true non-occurrence. However, we note that many of these cases will exist and adds to the challenging nature of primary care data.

B Pre-training performance evaluation

B.1 Representation Learning and demographic bias

During pre-training SurvivEHR learns to produce representations that summarise patient histories. Here we explore this concept space. Using patients from the pre-training cohort, we explore each patient's timeline up to each diagnosis they experienced. For example, given a patient who has experienced diagnoses of anxiety, depression, and arthritis, we extract three trajectories up to and including each of these events.

First, we explore the effect of static covariates on the latent embedding. In Supplementary Figure S3 we plot the UMAP latent embedding $\mathbf{T}_c$ up to and including each of their diagnosis events. In Supplementary Figures S3a–S3d, we label by different static covariates: sex, ethnicity, deprivation level given by their Index of Multiple Deprivation, and year of birth. The only clear associations were sex and birth year, as both will be significant predictive risk factors in most health conditions. Additionally, in Supplementary Figure S3e and Supplementary Figure S3f we plot the same embedding labelled by the number of historical morbidities, and the last seen diagnoses, respectively. For the former we see no clear pattern emerge. For the latter we see some emergent structure, including a clear cluster of eczema diagnoses that is neighboured by asthma diagnoses. However, this is explained by the birth year bias, where each can be explained by the age of the population in this area of the concept space. Additionally, we see a separate cluster of patients who died, but this is explained by this event marking the end of any patient records.

We continue by exploring the concept space of the same patients, but with the static covariate contributions $\mathbf{e}^{\text{static}}$ set to zero. These are shown in Supplementary Figure S4. As before, we plot the concept space labelled by the number of morbidities and the last seen diagnosis. Here we again see no clear structure when labelled by the number of historical morbidities. However, when labelled by last seen diagnosis we see some further structure emerge. However, we note that there remains a lack of distinctive sub-structures.

B.2 SurvivEHR captures known clinical associations

Through the typical recursive strategy of predicting the next event, SurvivEHR is capable of forecasting future clinical trajectories. Taking a population of patients, we present SurvivEHR with each held-out patient's complete history and ask it to predict the next three clinical events. We then analyse the conditional transition matrix that captures how often events arise together. This is given by

$$P(\text{next} = i \mid \text{prior} = j) = \frac{N_{j \to i}}{\sum_k N_{j \to k}},$$

where $N_{j \to i}$ denotes the number of observed transitions from event j to event i.

For the pre-training cohort, the resulting transition matrices are shown in Supplementary Figures S5–S7 for observed transitions (A) from diagnoses to diagnoses; (B) from diagnosis to medication; and (C) from investigations to diagnosis. Cells based on less than 5 observations or conditional probabilities less than 0.01 are masked for clarity.

References

[1] Krishna Margadhamane Gokhale et al. "Data extraction for epidemiological research (DExtER): a novel tool for automated clinical epidemiology studies". In: *European journal of epidemiology* 36.2 (2021), pp. 165–178.

[2] Ted Dunning and Otmar Ertl. "Computing extremely accurate quantiles using t-digests". In: *arXiv preprint arXiv:1902.04023* (2019).

Tables

Supplementary Table S1: SurvivEHR event vocabulary. Each event category is composed of a collection of medical codes which are collated under the same token. Some categories, such as those recorded under different units, are combined for clarity during plotting.

Event category	Plotting label	Token
PAD	-	0
UNK	-	1
ADDISONS_DISEASE	Addison's	2
ADDISON_DISEASE	Addison's	6
AF	AF	81
ALCOHOLMISUSE_V2	Alcohol misuse	94
ALLCANCER_NOHAEM_NOBCC	Cancer	104
ALLERGICRHINITISCONJ	Allergic rhinitis	123
ALL_DEMENTIA	Dementia	75
ANXIETY	Anxiety	126
ANY_DEAFNESS_HEARING_LOSS_V2	Hearing loss	114
AORTICANEURYSM_V2	Aortic aneurysms	30
ASTHMA_PUSHASTHMA	Asthma	134
ATOPICECZEMA	Eczema	136
AUTISM	Autism	39
BIPOLAR	BPAD	31
BRONCHIECTASIS	Bronchiectasis	32
CHRONICFATIGUESYNDROMEMM_V2	CFS	26
CHRONIC_LIVER_DISEASE_ALCOHOL	Alcoholic liver disease	19
CKDSTAGE3TO5	CKD	93
COPD	COPD	83
CROHNS_DISEASE	CD	23
CYSTICFIBROSIS	CF	3
DEATH	Death	106
DEPRESSION	Depression	133
DOWNSSYNDROME	Down syndrome	7
EATINGDISORDERS	Eating disorders	44
ENDOMETRIOSIS_ADENOMYOSIS_V2	Endometriosis	48
EPILEPSY	Epilepsy	60
FIBROMYALGIA	Fibromyalgia	37
GOUT	Gout	79
HAEMOCHROMATOSIS_V2	Haemochromatosis	8
HF_V3	CCF	74
HIVAIDS	HIV	12
HYPERTENSION	Hypertension	129
HYPERTHYROIDISM_V2	Hyperthyroidism	49
HYPOTHYROIDISM_DRAFT_V1	Hypothyroidism	91
IHDINCLUDINGMI_OPTIMALV2	IHD	95
ILD_SH	ILD	18
ISCHAEMICSTROKE_V2	Ischaemic stroke	41
LEUKAEMIA_PREVALENCEV2	Leukaemia	16
LYMPHOMA_PREVALENCE_V2	Lymphoma	22
MENIERESDISEASE	Ménière's	21
MINFARCTION	MI	67
MS	MS	14
NAFLD_V2	MAFLD	40
OSA	OSA	52

Continued on next page

Supplementary Table S1 – continued from previous page

Event category	Plotting label	Token
OSTEOARTHRITIS	OA	120
OSTEOPOROSIS	Osteoporosis	78
OTHER_CHRONIC_LIVER_DISEASE_OPTIMAL	Other liver disease	57
PAD_STRICT	PAD	56
PARKINSONS	PD	29
PERIPHERAL_NEUROPATHY	Peripheral neuropathy	89
PERNICIOUSANAEMIA	Pernicious anemia	20
PLASMACELL_NEOPLASM_V2	Myeloma	9
PMRANDGCA	PMR	45
POLYCYSTIC_OVARIAN_SYNDROME_PCOS_V2	PCOS	58
PREVALENT_IBS_V2	IBS	97
PSORIASIS	Psoriasis	82
PSORIATICARTHRITIS2021	Psoriatic arthritis	13
PTSDDIAGNOSIS	PTSD	35
PVD_V3	PVD	43
RHEUMATOIDARTHRITIS	RA	46
SCHIZOPHRENIAMM_V2	Schizophrenia	34
SICKLE_CELL_DISEASE_V2	Sickle cell	5
SJOGRENSSYNDROME	Sjögren’s	10
STROKEUNSPECIFIED_V2	Other stroke	65
STROKE_HAEMRGIC	Haemorrhagic stroke	28
SUBSTANCEMISUSE	Drug misuse	70
SYSTEMIC_LUPUS_ERYTHEMATOSUS	SLE	11
SYSTEMIC_SCLEROSIS	Systemic sclerosis	4
TYPE1DM	T1DM	36
TYPE2DIABETES	T2DM	100
ULCERATIVE_COLITIS	UC	33
VALVULARDISEASES_V2	Valvulopathy	62
VISUAL_IMPAIRMENT	Visual impairment	38
ACE_Inhibitors_D2T	ACEI	259
AMD_DH_Donepezil	Donepezil	142
AMD_DH_Memantine	Memantine	116
ARBs_Luyuan	ARB	221
Acarbose_AURUM	Acarbose	72
AldosteroneAntagonist_D2T	Aldosterone antagonist	155
AllHIVdrugs_HIV	Antiretrovirals	24
All_AntiArrhythmics_D2T	Antiarrhythmics	191
All_Antiplatelets	Antiplatelet	256
All_Diuretics_D2T	Diuretic	257
All_Diuretics_ExclLactones_D2T	Diuretics (excl. lactones)	258
AlphaBlocker	Alpha blocker	210
Amantadine	Amantadine	68
Amitriptyline_optimal	Amitriptyline	205
Anticonvulsants_OPTIMAL	Anticonvulsants	233
Antihistamines_second_generation_H1_antagonists	Antihistamine	207
Antipsychotics_OPTIMAL	Antipsychotics	196
Anxiolytics_mumpredict	Anxiolytics	198
Aspirin_OPTIMAL	Aspirin	253
Benzodiazepines	Benzodiazepines	213
BetaBlockers_OPTIMAL	Beta blocker	228
Bisphosphonates_osteoporosis	Bisphosphonates	195
COCP_reg_contraception	COCP	201

Continued on next page

Supplementary Table S1 – continued from previous page

Event category	Plotting label	Token
COMT_Optimal	COMT inhibitor	90
CalciumChannelBlck_D2T	CCB	250
Calcium_supplements_Optimal	Calcium supplement	216
Carbamazepine_Optimal	Carbamazepine	158
DPP4inhibitors_OPTIMAL	DPP4 inhibitor	156
Dementia	Dementia	151
Digoxin_	Digoxin	174
Direct_Acting_Oral_Anticoagulants_DOAC	DOAC	164
Fibrate_FenofibrateOnly	Fenofibrate	115
First_gen_H1_antihistamines	Antihistamine	171
GLP_1Aurum	GLP1 agonist	121
Gabapentin_Oral_OPTIMAL	Gabapentin	175
Galantamine_NEW	Galantamine	88
HydroxychloroquineAURUM_Zhaonan	Hydroxychloroquine	124
ICS_PUSHAsthma	Inhaled steroids	242
Insulin_Aurum_v2	Insulin	188
LABA_PUSH_Asthma	LABA	166
LAMA_PUSHAsthma	LAMA	169
LKA_PUSHAsthma	Montelukast	149
LeflunomideAURUM_Zhaonan	Leflunomide	76
Levothyroxine_	Levothyroxine	252
Lipid_lowering_drugs_Optimal	Lipid lowering drug	264
Lithium_OPTIMAL	Lithium	137
MAO_B_Optimal	MAOIs	87
Meglitinides_Aurum	Meglitinides	69
Metformin_612_A10BD2	Metformin	224
MethotrexateAURUM_Zhaonan	Methotrexate	147
Mirtazapine	Mirtazapine	182
Monoamine_Oxidase_Inhibitors_OPTIMAL	MAOIs	53
NSAIDS_oral_OPTIMAL_final	NSAID	260
Nasal_steroids_optimal	Nasal steroid	189
OCS_PUSHAsthma2	Oral steroids	192
POP_reg_contraceptive	POP	157
Paracetamol_OPTIMAL	Paracetamol	230
Pregabalin_Optimal	Pregabalin	162
PropranololAnxiety_mumpredict	Propanolol	160
Rivastigmine	Rivastigmine	92
SABA_PUSHAsthma	SABA	234
SGLT2inhibitors_Optimal	SGLT2 inhibitor	131
SNRIs_OPTIMAL	SNRI	178
SSRIs_Optimal	SSRI	254
Statins	Statin	263
Strong_Opioids_2022_Final	Strong opiates	197
SulfasalazineAURUM_Zhaonan	Sulfasalazine	132
Sulphonylureas_Aurum	Sulphonylureas	200
Systemic_oral_corticosteroids_optimal	Oral steroids	199
Thiazide_Diuretics_v2	Thiazides	249
Thiazolidinediones_Aurum_JC	TZDs	143
Tricyclic_Antidepressants_final	TCA	218
UricAcid_LoweringDrugs_optimal	Allopurinol	177
ValproateMigraine	Sodium valproate	161
Vitamin_D	Vitamin D supplement	222

Continued on next page

Supplementary Table S1 – continued from previous page

Event category	Plotting label	Token
Warfarin_	Warfarin	203
Weak_Opioids_Final	Weak opiate	255
all_contraceptive	Contraceptives	211
asthma_combined_inhalers	Combination inhaler	190
levodopa_Optimal	Levodopa	148
phosphodiesterasetype5inhibitor_OPTIMAL	Phosphodiesterase inhibitor	167
25_Hydroxyvitamin_D2_level_92	Vitamin D	84
25_Hydroxyvitamin_D3_level_90	Vitamin D	86
AST___aspartate_transam_SGOT__46	AST	107
AST_serum_level_47	AST	154
Albumin___creatinine_ratio_37	urine ACR	42
Basophil_count_22	Basophils	231
Blood_calcium_level_38	Calcium	63
Blood_urea_28	Urea	85
Body_mass_index_3	BMI	247
Brain_natriuretic_peptide_level_66	BNP	51
Calcium_adjusted_level_41	Calcium	103
Calculated_LDL_cholesterol_level_103	LDL	146
Combined_total_vitamin_D2_and_D3_level_93	Vitamin D	66
Corrected_serum_calcium_level_42	Calcium	172
Current_smoker_83	Smoker	152
Diastolic_blood_pressure_5	DBP	261
Eosinophil_count_21	Eosinophils	235
Erythrocyte_sedimentation_rate_61	ESR	193
Ex_smoker_84	Ex-smoker	184
Free_T4_level_76	T4	101
GFR_calculated_abbreviated_MDRD_34	eGFR	223
Haematocrit___PCV_16	Haematocrit	135
Haematocrit_15	Haematocrit	226
Haemoglobin_A1c_level___IFCC_standardised_6	HbA1c	202
Haemoglobin_A1c_level_8	HbA1c	145
Haemoglobin_estimation_9	Haemoglobin	244
HbA1c_level__DCCT_aligned__7	HbA1c	165
INR___international_normalised_ratio_81	INR	64
International_normalised_ratio_82	INR	185
Lymphocyte_count_20	Lymphocytes	238
Mean_corpusc_Hb_conc__MCHC__14	MCHC	220
Mean_corpusc_haemoglobin_MCH__13	MCHC	232
Mean_corpuscular_volume__MCV__11	MCV	240
Monocyte_count_23	Monocytes	237
N_terminal_pro_brain_natriuretic_peptide_level_67	BNP	17
Neutrophil_count_19	Neutrophils	239
Never_smoked_tobacco_85	Never smoker	217
Non_HDL_cholesterol_level_108	Non-HDL	118
O_E___height_1	Height	219
O_E___weight_2	Weight	251
Plasma_B_natriuretic_peptide_level_69	BNP	25
Plasma_C_reactive_protein_60	CRP	150
Plasma_HDL_cholesterol_level_101	HDL	111
Plasma_LDL_cholesterol_level_104	LDL	102
Plasma_N_terminal_pro_B_type_natriuretic_peptid...	BNP	15

Continued on next page

Supplementary Table S1 – continued from previous page

Event category	Plotting label	Token
Plasma_TSH_level_73	TSH	110
Plasma_alanine_aminotransferase_level_44	ALT	130
Plasma_albumin_level_52	Albumin	128
Plasma_alkaline_phosphatase_level_49	ALP	122
Plasma_calcium_level_40	Calcium	99
Plasma_cholesterol_HDL_ratio_96	Cholesterol: HDL ratio	77
Plasma_corrected_calcium_level_43	Calcium	96
Plasma_creatinine_level_32	Creatinine	140
Plasma_ferritin_level_62	Ferritin	50
Plasma_free_T4_level_77	T4	80
Plasma_gamma_glutamyl_transferase_level_58	GGT	108
Plasma_potassium_level_27	Potassium	138
Plasma_pro_brain_natriuretic_peptide_level_64	BNP	27
Plasma_sodium_level_25	Sodium	139
Plasma_total_bilirubin_level_54	Bilirubin	125
Plasma_total_cholesterol_level_99	Total cholesterol	117
Plasma_triglyceride_level_106	Triglycerides	105
Plasma_urea_level_30	Urea	109
Platelet_count_12	Platelets	243
Red_blood_cell__RBC__count_10	RBC	236
Red_blood_cell_distribution_width_17	RDW	208
Serum_25_Hydroxy_vitamin_D3_level_88	Vitamin D	98
Serum_C_reactive_protein_level_59	CRP	176
Serum_HDL_cholesterol_level_100	HDL	209
Serum_LDL_cholesterol_level_102	LDL	194
Serum_N_terminal_pro_B_type_natriuretic_peptide...	BNP	61
Serum_T4_level_78	T4	73
Serum_TSH_level_71	TSH	214
Serum_alanine_aminotransferase_level_45	AST	206
Serum_albumin_51	Albumin	229
Serum_alkaline_phosphatase_50	ALP	225
Serum_bilirubin_level_53	Bilirubin	183
Serum_calcium_39	Calcium	186
Serum_cholesterol_97	Cholesterol	215
Serum_cholesterol_HDL_ratio_94	Cholesterol: HDL ratio	187
Serum_creatinine_31	Creatinine	248
Serum_ferritin_63	Ferritin	179
Serum_folate_80	Folic acid	163
Serum_free_T4_level_75	T4	180
Serum_gamma_glutamyl_transferase_level_57	GGT	181
Serum_non_high_density_lipoprotein_cholesterol_...	Non-HDL	153
Serum_potassium_26	Potassium	245
Serum_pro_brain_natriuretic_peptide_level_65	BNP	54
Serum_sodium_24	Sodium	246
Serum_total_25_hydroxy_vitamin_D_level_87	Vitamin D	112
Serum_total_bilirubin_level_56	Bilirubin	212
Serum_total_cholesterol_level_98	Cholesterol	141
Serum_triglycerides_105	Triglycerides	204
Serum_urea_level_29	Urea	227
Serum_vitamin_B12_79	Vitamin B12	173
Serum_vitamin_D2_level_89	Vitamin D	55
Serum_vitamin_D_86	Vitamin D	119

Continued on next page

Supplementary Table S1 – continued from previous page

Event category	Plotting label	Token
Systolic_blood_pressure_4	SBP	262
TSH___thyroid_stim_hormone_72	TSH	127
TSH_level_74	TSH	71
Total_25_hydroxyvitamin_D_level_91	Vitamin D	59
Total_alkaline_phosphatase_48	ALP	144
Total_bilirubin_55	Bilirubin	113
Total_cholesterol_HDL_ratio_95	Cholesterol: HDL ratio	170
Total_white_cell_count_18	WCC	241
Urine_albumin_creatinine_ratio_35	Urine ACR	168
Urine_microalbumin_creatinine_ratio_36	Urine ACR	47
eGFR_using_creatinine_CKD_EPI_per_1_73_square_m...	eGFR	159

Supplementary Table S2: Event counts. Number of clinical records used for pre-training, collected from over 26.5 million patients across 1478 practices in England. Counts are extracted from the SQLite database, and the count of observed values indicates the number available before outlier removal.

Event type	Number of events	Token count	Count of observed values
Diagnoses	74	51,003,640	n/a
Medications	81	3,970,984,804	n/a
Investigations	108	3,533,426,831	3,364,437,065
**Total**	263	7.6 Billion	3.4 Billion

Supplementary Table S3: Summary statistics of diagnoses. Summary statistics of raw diagnosis data extracted from CPRD by DExtER. These are calculated before any pre-processing has been performed.

Event category	Count
ADDISONS_DISEASE	6691
ADDISON_DISEASE	11794
AF	731332
ALCOHOLMISUSE_V2	1125212
ALLCANCER_NOHAEM_NOBCC	1496973
ALLERGICRHINITISCONJ	3291165
ALL_DEMENTIA	528602
ANXIETY	3560978
ANY_DEAFNESS_HEARING_LOSS_V2	2282766
AORTICANEURYSM_V2	101134
ASTHMA_PUSHASTHMA	4175115
ATOPICECZEMA	4369082
AUTISM	156860
BIPOLAR	108852
BRONCHIECTASIS	112618
CHRONICFATIGUESYNDROMEMM_V2	82799
CHRONIC_LIVER_DISEASE_ALCOHOL	63405
CKDSTAGE3TO5	1088754
COPD	751320
CROHNS_DISEASE	81250
CYSTICFIBROSIS	7053
DEATH	1629100

Continued on next page

Supplementary Table S3 – continued from previous page

Event category	Count
DEPRESSION	4109336
DOWNSSYNDROME	17006
EATINGDISORDERS	191873
ENDOMETRIOSIS_ADENOMYOSIS_V2	209157
EPILEPSY	377341
FIBROMYALGIA	153213
GOUT	632089
HAEMOCHROMATOSIS_V2	18631
HF_V3	524982
HIVAIDS	41951
HYPERTENSION	3934473
HYPERTHYROIDISM_V2	217964
HYPOTHYROIDISM_DRAFT_V1	932079
IHDINCLUDINGMI_OPTIMALV2	1162843
ILD_SH	58104
ISCHAEMICSTROKE_V2	178437
LEUKAEMIA_PREVALENCEV2	54438
LYMPHOMA_PREVALENCE_V2	80511
MENIERESDISEASE	73688
MINFARCTION	477556
MS	53204
NAFLD_V2	158688
OSA	234938
OSTEOARTHRITIS	2653242
OSTEOPOROSIS	628118
OTHER_CHRONIC_LIVER_DISEASE_OPTIMAL	289252
PAD_STRICT	254632
PARKINSONS	91718
PERIPHERAL_NEUROPATHY	840638
PERNICIOUSANAEMIA	73331
PLASMACELL_NEOPLASM_V2	20301
PMRANDGCA	195907
POLYCYSTIC_OVARIAN_SYNDROME_PCOS_V2	332297
PREVALENT_IBS_V2	1210229
PSORIASIS	743897
PSORIATICARTHRITIS2021	51273
PTSDDIAGNOSIS	126269
PVD_V3	185904
RHEUMATOIDARTHRITIS	197253
SCHIZOPHRENIAMM_V2	125141
SICKLE_CELL_DISEASE_V2	11159
SJOGRENSSYNDROME	23326
STROKEUNSPECIFIED_V2	446046
STROKE_HAEMRGIC	83609
SUBSTANCEMISUSE	502552
SYSTEMIC_LUPUS_ERYTHEMATOSUS	26820
SYSTEMIC_SCLEROSIS	8772
TYPE1DM	145143
TYPE2DIABETES	1404325
ULCERATIVE_COLITIS	120361
VALVULARDISEASES_V2	401061
VISUAL_IMPAIRMENT	155707

Supplementary Table S4: Summary statistics of medications. Summary statistics of raw medication data extracted from CPRD by DExtER. These are calculated before any pre-processing has been performed.

Event category	Count
ACE_Inhibitors_D2T	191462111
AMD_DH_Donepezil	5635153
AMD_DH_Memantine	2309471
ARBs_Luyuan	72329278
Acarbose_AURUM	507434
AldosteroneAntagonist_D2T	11182704
AllHIVdrugs_HIV	82262
All_AntiArrhythmics_D2T	29502352
All_Antiplatelets	162940629
All_Diuretics_D2T	176070847
All_Diuretics_ExclLactones_D2T	176070847
AlphaBlocker	51635379
Amantadine	479791
Amitriptyline_optimal	46415710
Anticonvulsants_OPTIMAL	90774779
Antihistamines_second_generation_H1_antagonists	47308115
Antipsychotics_OPTIMAL	31658166
Anxiolytics_mumpredict	32874923
Aspirin_OPTIMAL	131327634
Benzodiazepines	55608827
BetaBlockers_OPTIMAL	79374522
Bisphosphonates_osteoporosis	30294675
COCP_reg_contraception	34316525
COMT_Optimal	886259
CalciumChannelBlck_D2T	108721302
Calcium_supplements_Optimal	57542223
Carbamazepine_Optimal	12812897
DPP4inhibitors_OPTIMAL	12000147
Dementia	9738544
Digoxin_	17497910
Direct_Acting_Oral_Anticoagulants_DOAC	14815747
Fibrate_FenofibrateOnly	2305308
First_gen_H1_antihistamines	16181607
GLP_1Aurum	2676211
Gabapentin_Oral_OPTIMAL	17508445
Galantamine_NEW	830429
HydroxychloroquineAURUM_Zhaonan	3312310
ICS_PUSHAsthma	94873269
Insulin_Aurum_v2	28641496
LABA_PUSH_Asthma	14958767
LAMA_PUSHAsthma	15239995
LKA_PUSHAsthma	8537907
LeflunomideAURUM_Zhaonan	586799
Levothyroxine_	117844926
Lipid_lowering_drugs_Optimal	263876709
Lithium_OPTIMAL	4395155
MAO_B_Optimal	829174
Meglitinides_Aurum	486644
Metformin_612_A10BD2	74162080
MethotrexateAURUM_Zhaonan	7884622

Continued on next page

Supplementary Table S4 – continued from previous page

Event category	Count
Mirtazapine	23524446
Monoamine_Oxidase_Inhibitors_OPTIMAL	236966
NSAIDS_oral_OPTIMAL_final	219183349
Nasal_steroids_optimal	29010523
OCS_PUSHAsthma2	29568929
POP_reg_contraceptive	12186107
Paracetamol_OPTIMAL	85242874
Pregabalin_Optimal	14497547
PropranololAnxiety_mumpredict	12924874
Rivastigmine	963471
SABA_PUSHAsthma	91182585
SGLT2inhibitors_Optimal	4059940
SNRIs_OPTIMAL	21379065
SSRIs_Optimal	132417423
Statins	249911634
Strong_Opioids_2022_Final	31728471
SulfasalazineAURUM_Zhaonan	4084855
Sulphonylureas_Aurum	34289555
Systemic_oral_corticosteroids_optimal	33516650
Thiazide_Diuretics_v2	105228802
Thiazolidinediones_Aurum_JC	6191596
Tricyclic_Antidepressants_final	68893019
UricAcid_LoweringDrugs_optimal	20559558
ValproateMigraine	13594697
Vitamin_D	72657845
Warfarin_	37353354
Weak_Opioids_Final	140622354
all_contraceptive	53655141
asthma_combined_inhalers	29469516
levodopa_Optimal	8483033
phosphodiesterasetype5inhibitor_OPTIMAL	15057609

Supplementary Table S5: Summary statistics of investigations. Summary statistics of raw investigation data extracted from CPRD by DExtER. These are calculated before any pre-processing has been performed.

Event category	Mean	Min	Max	Count	# Missing
25_Hydroxyvitamin_D2_level_92	3.91	0	686	782791	89321
25_Hydroxyvitamin_D3_level_90	47.1	0	952	809104	27986
AST___aspartate_transam_SGOT__46	26.6	0	1.5e+04	1738489	57876
AST_serum_level_47	27.3	-5.00	2.1e+04	10837982	352631
Albumin___creatinine_ratio_37	10.7	-1.00	1.3e+04	180911	102491
Basophil_count_22	0.0501	-0.100	1.1e+05	86869779	1227239
Blood_calcium_level_38	2.35	0	440	415717	30253
Blood_urea_28	6.51	0	1.3e+03	785766	113905
Body_mass_index_3	293	-3.3e+04	2.1e+09	99868822	2109510
Brain_natriuretic_peptide_level_66	417	0	5e+05	229202	69884
Calcium_adjusted_level_41	2.37	0	279	1464226	6315
Calculated_LDL_cholesterol_level_103	2.82	-8.40	240	6967780	248034
Combined_total_vitamin_D2_and_D3_level_93	52.4	0	1.2e+03	452984	28416

Continued on next page

Event category	Mean	Min	Max	Count	# Missing
Corrected_serum_calcium_level_42	3.89	-2.58	1.2e+07	16683796	419151
Current_smoker_83	69.8	-2e+03	1.7e+07	10592787	8083175
Diastolic_blood_pressure_5	3.8e+03	-120	9.1e+11	241026671	301816
Eosinophil_count_21	0.234	-0.600	4.4e+05	92193433	584466
Erythrocyte_sedimentation_rate_61	18.4	-20.0	1.3e+07	29892785	5233008
Ex_smoker_84	410	-2e+03	2e+08	24891632	22903077
Free_T4_level_76	2.9e+05	-134	9.8e+10	1405341	150687
GFR_calculated_abbreviated_MDRD_34	72.0	-90.0	1.8e+07	73709249	7876443
Haematocrit___PCV_16	5.57	-0.465	9.4e+03	4346108	32185
Haematocrit_15	3.78	0	2.1e+05	76582325	287307
Haemoglobin_A1c_level___IFCC_standard...	1.9e+05	-79.0	6e+12	35091904	2549714
Haemoglobin_A1c_level_8	13.5	0	1e+06	6516701	4237674
Haemoglobin_estimation_9	79.6	-112	1.4e+07	97391693	858067
HbA1c_level__DCCT_aligned__7	11.7	-41.0	3.3e+07	14948615	2828072
INR___international_normalised_ratio_81	2.78	0	3.1e+04	426165	16990
International_normalised_ratio_82	23.9	-34.0	3e+07	27966697	3393266
Lymphocyte_count_20	365	-35.0	3.7e+09	92896401	526313
Mean_corpusc_Hb_conc__MCHC__14	186	-295	3.5e+05	70867102	242847
Mean_corpusc_haemoglobin_MCH__13	82.8	-28.9	3.6e+07	90498376	277562
Mean_corpuscular_volume__MCV__11	2.1e+04	-100	2e+12	94723195	313276
Monocyte_count_23	81.9	-4.00	3.7e+09	92489278	510098
N_terminal_pro_brain_natriuretic_peptide_level_67	760	0	7.5e+04	57470	4069
Neutrophil_count_19	85.1	-67.0	3.7e+09	93489124	637721
Never_smoked_tobacco_85	5.09	-25.0	2e+03	60751822	59213176
Non_HDL_cholesterol_level_108	3.45	-1.60	3.4e+03	2408310	14410
O_E___height_1	664	-1.1e+05	2e+10	69089436	336366
O_E___weight_2	5.8e+04	-1.2e+04	4e+12	117401338	589408
Plasma_B_natriuretic_peptide_level_69	215	0	5.7e+05	82488	3711
Plasma_C_reactive_protein_60	13.7	-3.8e+03	1.3e+07	9155551	1754490
Plasma_HDL_cholesterol_level_101	1.8e+03	0	3.7e+09	2173456	61682
Plasma_LDL_cholesterol_level_104	2.84	0	502	1434461	184941
Plasma_N_terminal_pro_B_type_natriuretic_peptid...	832	0.200	1e+05	53534	1229
Plasma_TSH_level_73	2.59	-7.80	2.5e+04	2049380	32179
Plasma_alanine_aminotransferase_level_44	26.8	0	1.1e+04	4012790	23486
Plasma_albumin_level_52	40.8	0	4.4e+04	3719377	11968
Plasma_alkaline_phosphatase_level_49	128	-24.0	2e+04	3290802	9406
Plasma_calcium_level_40	2.38	0	378	1294409	7535
Plasma_cholesterol_HDL_ratio_96	3.79	0	999	587483	11890
Plasma_corrected_calcium_level_43	2.35	0	248	1168224	268132
Plasma_creatinine_level_32	915	0	3.7e+09	4513054	23789
Plasma_ferritin_level_62	84.2	0	2.5e+04	220380	52482
Plasma_free_T4_level_77	5.7e+05	-77.2	2.5e+10	724108	42413
Plasma_gamma_glutamyl_transferase_level_58	53.6	0	1.2e+04	1762750	13310
Plasma_potassium_level_27	849	0	3.7e+09	4445152	42312
Plasma_pro_brain_natriuretic_peptide_level_64	550	0	6.7e+04	83343	13214
Plasma_sodium_level_25	980	0	3.7e+09	4458632	33102
Plasma_total_bilirubin_level_54	11.0	0	1.1e+04	3443114	13631
Plasma_total_cholesterol_level_99	1.6e+03	0	3.7e+09	2331589	28898
Plasma_triglyceride_level_106	2.4e+03	0	3.7e+09	1573268	24409
Plasma_urea_level_30	5.96	0	808	1991274	22825
Platelet_count_12	2.5e+04	-560	3.7e+09	95904116	521873
Red_blood_cell__RBC__count_10	6.1e+04	-4.46	3.7e+09	92326200	470623

Continued on next page

Supplementary Table S5 – continued from previous page

Event category	Mean	Min	Max	Count	# Missing
Red_blood_cell_distribution_width_17	14.0	0	2.6e+05	49751137	3364137
Serum_25_Hydroxy_vitamin_D3_level_88	50.0	0	2e+04	1211141	363513
Serum_C_reactive_protein_level_59	23.8	-115	2.1e+08	19234068	2893243
Serum_HDL_cholesterol_level_100	1.51	-39.0	1.8e+06	50953183	936362
Serum_LDL_cholesterol_level_102	387	-1e+06	3.7e+09	30073152	981676
Serum_N_terminal_pro_B_type_natriuretic_peptide...	786	0	1.2e+05	383790	48134
Serum_T4_level_78	5.2e+06	-25.0	1.5e+11	524777	73789
Serum_TSH_level_71	15.4	-21.4	2e+07	55655662	3197511
Serum_alanine_aminotransferase_level_45	27.1	-43.0	2.1e+05	46896239	777411
Serum_albumin_51	791	-45.0	9e+09	83105055	349161
Serum_alkaline_phosphatase_50	89.3	-165	1.5e+07	76052242	342655
Serum_bilirubin_level_53	16.2	-1.9e+05	1.2e+08	24090337	147279
Serum_calcium_39	2.64	-2.12	7e+06	28049902	1120505
Serum_cholesterol_97	74.7	-75.0	3.7e+09	57393841	2256817
Serum_cholesterol_HDL_ratio_94	137	-3.40	3.7e+09	28586515	637258
Serum_creatinine_31	535	-4.2e+04	3.7e+09	102915170	1314960
Serum_ferritin_63	120	-2.8e+06	1.9e+08	21412371	2581319
Serum_folate_80	8.60	-20.0	2.4e+04	14643748	1398479
Serum_free_T4_level_75	5.5e+05	0	1e+11	22579062	600152
Serum_gamma_glutamyl_transferase_level_57	57.4	-462	7e+04	22592131	788041
Serum_non_high_density_lipoprotein_cholesterol_...	3.40	-2.50	7.3e+03	10648096	27356
Serum_potassium_26	43.2	-2.2e+03	3.7e+09	99036754	1540838
Serum_pro_brain_natriuretic_peptide_level_65	701	0	7.7e+05	243565	47507
Serum_sodium_24	141	-140	6.9e+07	99409745	592495
Serum_total_25_hydroxy_vitamin_D_level_87	53.2	0	921	2178432	296091
Serum_total_bilirubin_level_56	32.0	-2.00	4.3e+07	54098383	344930
Serum_total_cholesterol_level_98	5.17	-7.60	1e+06	5185017	84358
Serum_triglycerides_105	445	-376	3.7e+09	42415036	434218
Serum_urea_level_29	5.8e+03	-24.1	2.4e+10	78670253	393889
Serum_vitamin_B12_79	715	-5.8e+08	3.7e+09	16797696	1665415
Serum_vitamin_D2_level_89	14.8	-5.00	728	249092	44373
Serum_vitamin_D_86	53.7	-39.7	3e+06	2478806	737039
Systolic_blood_pressure_4	601	-200	1.1e+11	241541988	318809
TSH___thyroid_stim_hormone_72	25.6	-50.0	8.1e+06	3568579	505048
TSH_level_74	4.04	-0.500	9.8e+03	507085	234183
Total_25_hydroxyvitamin_D_level_91	48.0	0	1.2e+03	333324	49869
Total_alkaline_phosphatase_48	84.5	0	9e+03	6461505	38583
Total_bilirubin_55	147	-7.00	1.8e+07	2193031	51422
Total_cholesterol_HDL_ratio_95	965	-3.10	3.7e+09	15760772	271673
Total_white_cell_count_18	250	-14.7	3.7e+09	94827537	648527
Urine_albumin_creatinine_ratio_35	996	-14.0	1e+10	15107807	4858601
Urine_microalbumin_creatinine_ratio_36	10.8	-5.80	3.2e+04	201318	107309
eGFR_using_creatinine_CKD_EPI_...	74.3	-90.0	8.7e+04	12869245	967096

Supplementary Table S6: Pre-processing bounds. Outlier and standardisation bounds. Values beyond these limits are treated as outliers and marked as missing by SurvivEHR. Remaining values are standardised between these bounds, centered at zero.

Event category	F_L	F_U
25_Hydroxyvitamin_D2_level_92	-4.67	10.9
25_Hydroxyvitamin_D3_level_90	-36.8	122
AST___aspartate_transam_SGOT__46	0.100	40000
AST_serum_level_47	0.100	40000
Albumin___creatinine_ratio_37	-4.32	8.83
Basophil_count_22	-0.0938	10.0
Blood_calcium_level_38	0.100	10.0
Blood_urea_28	0.100	11.1
Body_mass_index_3	5.00	130
Brain_natriuretic_peptide_level_66	-245	483
Calcium_adjusted_level_41	0.100	10.0
Calculated_LDL_cholesterol_level_103	0	30.0
Combined_total_vitamin_D2_and_D3_level_93	0.100	127
Corrected_serum_calcium_level_42	0.100	10.0
Current_smoker_83	0	80.0
Diastolic_blood_pressure_5	20.0	400
Eosinophil_count_21	-0.185	10.0
Erythrocyte_sedimentation_rate_61	0	49.7
Ex_smoker_84	0	80.0
Free_T4_level_76	0.100	1000
GFR_calculated_abbreviated_MDRD_34	0.100	300
Haematocrit___PCV_16	0.100	1.00
Haematocrit_15	0	1.00
Haemoglobin_A1c_level___IFCC_standardised_6	5.00	250
Haemoglobin_A1c_level_8	5.00	250
Haemoglobin_estimation_9	10.0	300
HbA1c_level__DCCT_aligned__7	5.00	250
INR___international_normalised_ratio_81	0.625	100
International_normalised_ratio_82	0.425	100
Lymphocyte_count_20	0.100	200
Mean_corpusc_Hb_conc__MCHC__14	10.0	500
Mean_corpusc_haemoglobin_MCH__13	1.00	100
Mean_corpuscular_volume__MCV__11	10.0	250
Monocyte_count_23	-0.0153	20.0
N_terminal_pro_brain_natriuretic_peptide_level_67	-609	1182
Neutrophil_count_19	0	50.0
Never_smoked_tobacco_85	0	0
Non_HDL_cholesterol_level_108	0	6.41
O_E___height_1	20.0	272
O_E___weight_2	0.250	650
Plasma_B_natriuretic_peptide_level_69	-179	368
Plasma_C_reactive_protein_60	-11.2	22.9
Plasma_HDL_cholesterol_level_101	0.343	3.90
Plasma_LDL_cholesterol_level_104	0	30.0
Plasma_N_terminal_pro_B_type_natriuretic_peptid...	-634	1243
Plasma_TSH_level_73	-1.34	500
Plasma_alanine_aminotransferase_level_44	0.100	40000
Plasma_albumin_level_52	2.00	180
Plasma_alkaline_phosphatase_level_49	1.00	281

Continued on next page

Supplementary Table S6 – continued from previous page

Event category	F_L	F_U
Plasma_calcium_level_40	0.100	10.0
Plasma_cholesterol_HDL_ratio_96	1.00	250
Plasma_corrected_calcium_level_43	0.100	1.00
Plasma_creatinine_level_32	1.00	140
Plasma_ferritin_level_62	-90.1	500000
Plasma_free_T4_level_77	0.100	1000
Plasma_gamma_glutamyl_transferase_level_58	-26.3	90.7
Plasma_potassium_level_27	0.100	10.0
Plasma_pro_brain_natriuretic_peptide_level_64	-347	670
Plasma_sodium_level_25	90.0	200
Plasma_total_bilirubin_level_54	-0.841	900
Plasma_total_cholesterol_level_99	1.64	50.0
Plasma_triglyceride_level_106	-0.488	3.46
Plasma_urea_level_30	0.100	10.1
Platelet_count_12	0	10000000
Red_blood_cell__RBC__count_10	0	500
Red_blood_cell_distribution_width_17	10.4	100
Serum_25_Hydroxy_vitamin_D3_level_88	0.100	124
Serum_C_reactive_protein_level_59	-8.03	17.4
Serum_HDL_cholesterol_level_100	0	3.90
Serum_LDL_cholesterol_level_102	0	30.0
Serum_N_terminal_pro_B_type_natriuretic_peptide...	-616	1198
Serum_T4_level_78	0.100	1000
Serum_TSH_level_71	-1.16	500
Serum_alanine_aminotransferase_level_45	0.100	40000
Serum_albumin_51	2.00	180
Serum_alkaline_phosphatase_50	1.00	20000
Serum_bilirubin_level_53	-0.918	900
Serum_calcium_39	0.100	10.0
Serum_cholesterol_97	1.76	50.0
Serum_cholesterol_HDL_ratio_94	1.00	250
Serum_creatinine_31	1.00	10000
Serum_ferritin_63	-113	500000
Serum_folate_80	-4.58	20.5
Serum_free_T4_level_75	0.100	10000
Serum_gamma_glutamyl_transferase_level_57	-29.0	94.8
Serum_non_high_density_lipoprotein_cholesterol_...	0	3.90
Serum_potassium_26	0.100	10.0
Serum_pro_brain_natriuretic_peptide_level_65	-500	931
Serum_sodium_24	90.0	200
Serum_total_25_hydroxy_vitamin_D_level_87	-19.2	124
Serum_total_bilirubin_level_56	-0.908	900
Serum_total_cholesterol_level_98	1.57	50.0
Serum_triglycerides_105	0	100
Serum_urea_level_29	0.100	10.2
Serum_vitamin_B12_79	5.00	20000
Serum_vitamin_D2_level_89	-7.75	20.6
Serum_vitamin_D_86	0.100	1000
Systolic_blood_pressure_4	30.0	400
TSH___thyroid_stim_hormone_72	-1.53	500
TSH_level_74	-1.96	500
Total_25_hydroxyvitamin_D_level_91	0.100	1000

Continued on next page

Supplementary Table S6 – continued from previous page

Event category	F_L	F_U
Total_alkaline_phosphatase_48	0.100	139
Total_bilirubin_55	-2.32	900
Total_cholesterol_HDL_ratio_95	1.00	250
Total_white_cell_count_18	0.100	5000
Urine_albumin_creatinine_ratio_35	-3.78	300
Urine_microalbumin_creatinine_ratio_36	-4.12	8.25
eGFR_using_creatinine_CKD_EPI_...	22.7	300

Component	Hyperparameter	Pre-training	Fine-tuning
Architecture	Layers	6	6
	Attention heads	6	6
	Hidden size	384	384
	Max sequence length	256	512
Context window	Repeated measurements	All	Last unique
	Global diagnoses	False	Append to context
Optimization	Batch size	64	512
	Epochs	10	20
	Early stopping	False	True
	Optimizer	AdamW	AdamW
	Backbone learning rate	3×10^{-4}	5×10^{-5}
	Head learning rate	3×10^{-4}	5×10^{-4}
Scheduler	Linear warmup steps	10,000	0
	Schedule	Cosine annealing	Reduce on plateau
	Warm restarts	True	False
	Warmup steps	10,000	-
	LR decay	0.8	-

Supplementary Table S7: Model hyperparameters used during pre-training and fine-tuning.

Case study	Stratification factor	Strata	C_{td} (↑)	IBS (↓)	INBLL (↓)
5-year hypertension risk	Gender	Male	0.817	0.0810	0.255
		Female	0.849	0.0685	0.217
	Ethnicity	White	0.834	0.0756	0.238
		Asian	0.830	0.0723	0.239
		Black	0.847	0.0855	0.265
		Mixed	0.817	0.138	0.419
		Other	0.848	0.0921	0.286
	Index of multiple deprivation	1	0.839	0.0782	0.246
		2	0.840	0.0732	0.231
		3	0.826	0.0744	0.235
		4	0.833	0.0737	0.235
		5	0.814	0.0940	0.287
	Number of existing morbidities	1	0.711	0.129	0.405
		2	0.819	0.0815	0.257
		3	0.855	0.0652	0.206
		4	0.861	0.0581	0.184
		5+	0.895	0.0471	0.150
5-year cardiovascular disease risk	Gender	Male	0.673	0.0371	0.153
		Female	0.708	0.0287	0.123
	Ethnicity	White	0.688	0.0326	0.137
		Asian	0.754	0.0316	0.129
		Black	0.726	0.0521	0.221
		Mixed	0.677	0.101	0.374
		Other	0.722	0.0912	0.316
	Index of multiple deprivation	1	0.670	0.0320	0.135
		2	0.697	0.0315	0.135
		3	0.654	0.0367	0.154
		4	0.702	0.0338	0.140
		5	0.651	0.0370	0.151
	Number of existing morbidities	1	0.702	0.0229	0.103
		2	0.680	0.0329	0.138
		3	0.676	0.0361	0.151
		4	0.689	0.0323	0.137
		5+	0.705	0.0393	0.158
5-year multi-morbidity risk	Gender	Male	0.652	0.149	0.453
		Female	0.662	0.147	0.447
	Ethnicity	White	0.656	0.149	0.452
		Asian	0.660	0.157	0.470
		Black	0.616	0.168	0.495
		Mixed	0.683	0.172	0.506
		Other	0.666	0.172	0.509
	Index of multiple deprivation	1	0.652	0.138	0.426
		2	0.657	0.143	0.437
		3	0.663	0.147	0.448
		4	0.649	0.158	0.476
		5	0.666	0.162	0.485
	Number of existing morbidities	0	0.671	0.124	0.388
		1	0.653	0.140	0.429
		2	0.649	0.149	0.453
		3	0.645	0.159	0.477
		4	0.639	0.167	0.498
		5+	0.637	0.178	0.525

Supplementary Table S8: Clinical risk prediction model performance stratified by demographics. The survival metrics (C_{td}, IBS and INBLL) evaluate the model's ability to predict the final outcome. We report these stratified by gender, ethnicity, the index of multiple deprivation (IMD) and the number of pre-existing morbidities (including the index diagnosis).

Figures

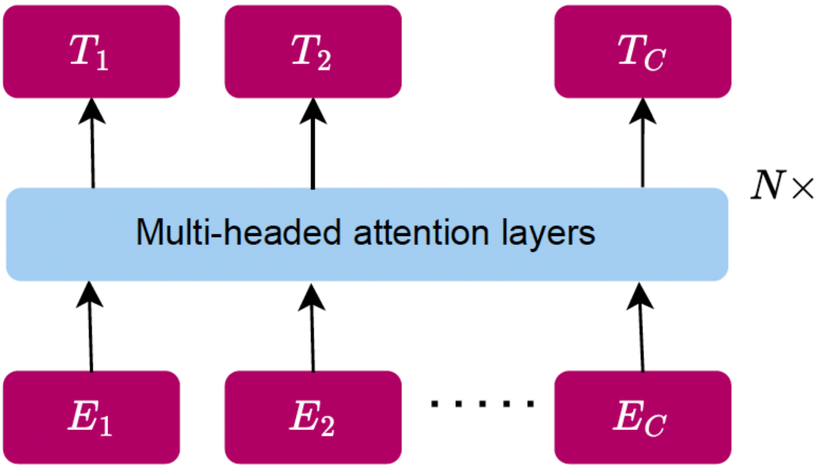

Supplementary Figure S1: Decoder architecture. SurvivEHR uses the Generative Pre-trained Transformer (GPT) decoder architecture to learn representations of a patient's prior health records. At any point in time all historic events $\mathbf{E}_1, \ldots \mathbf{E}_c$ can be processed to obtain a vector embedding that summarises the patient's history, $\mathbf{T}_c$.

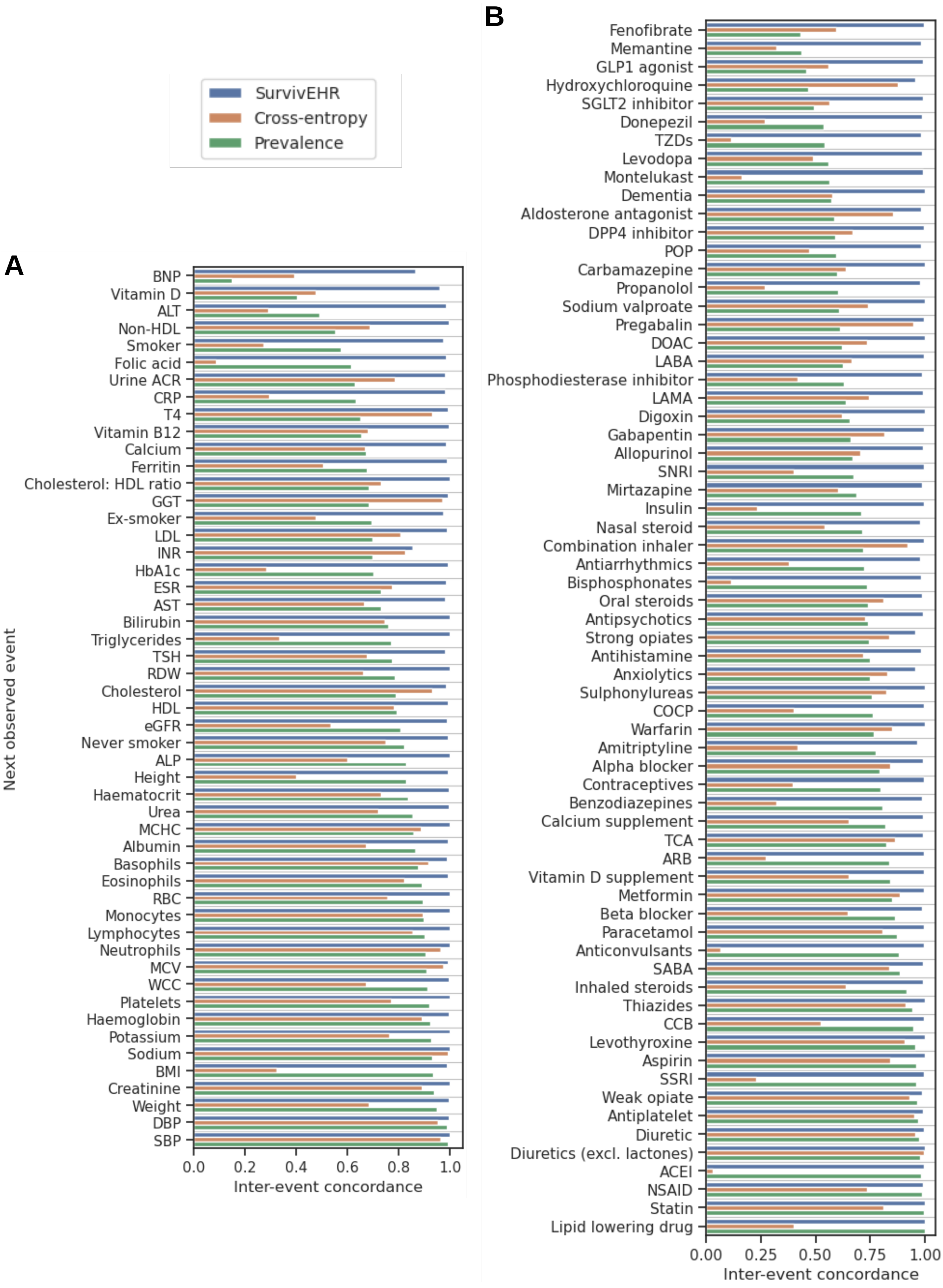

Supplementary Figure S2: Inter-event concordance. The inter-event concordance metric for each **A** investigation, and **B** medication. This quantifies the model's ability to correctly rank the risk of each diagnosis being the next-event a patient will experience across their lifetime. Some tokens are missing due to random sampling.

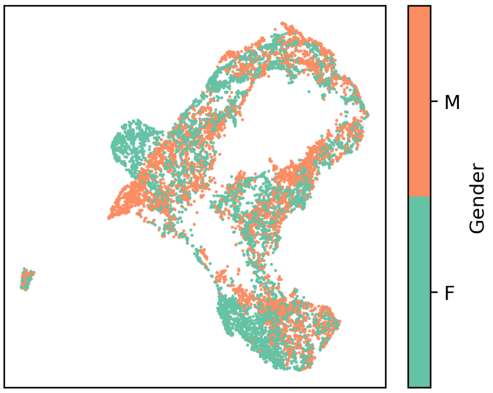

(a) Sex.

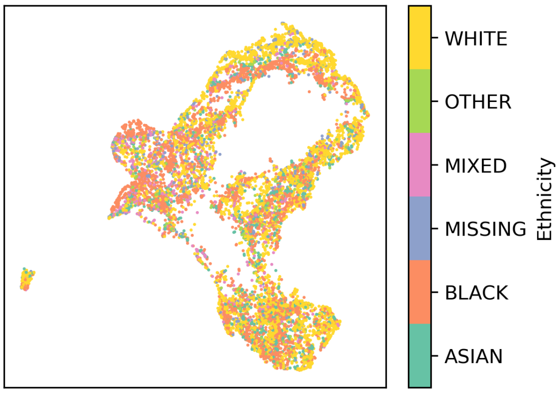

(b) Ethnicity.

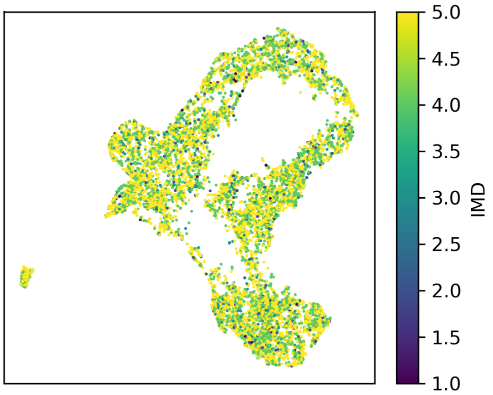

(c) Index of multiple deprivation.

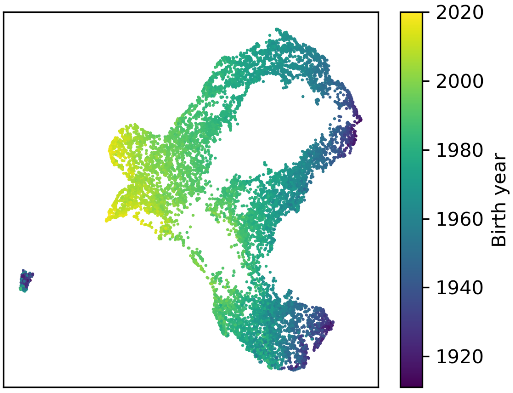

(d) Year of birth.

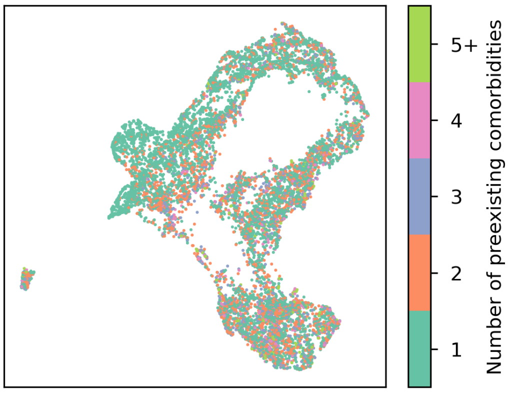

(e) Number of pre-existing conditions.

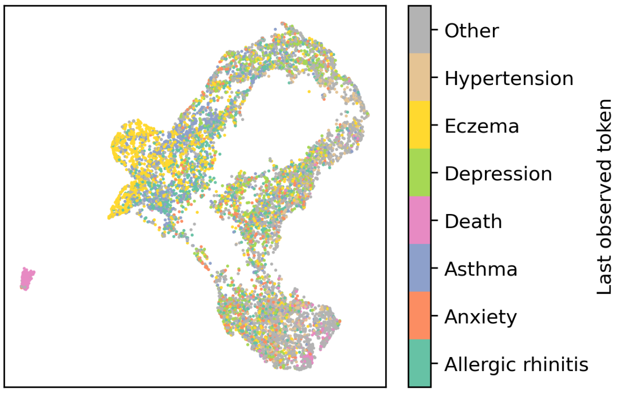

(f) Last observed diagnosis.

Supplementary Figure S3: Learnt representations. UMAP representations $\mathbf{T}_c$ of patient health records in the held-out pre-training cohort. We include histories up to and including each diagnosis a patient experiences.

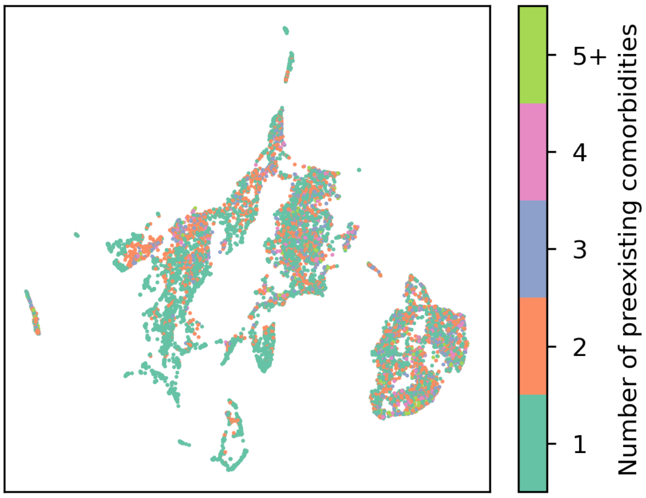

(a) Number of pre-existing conditions.

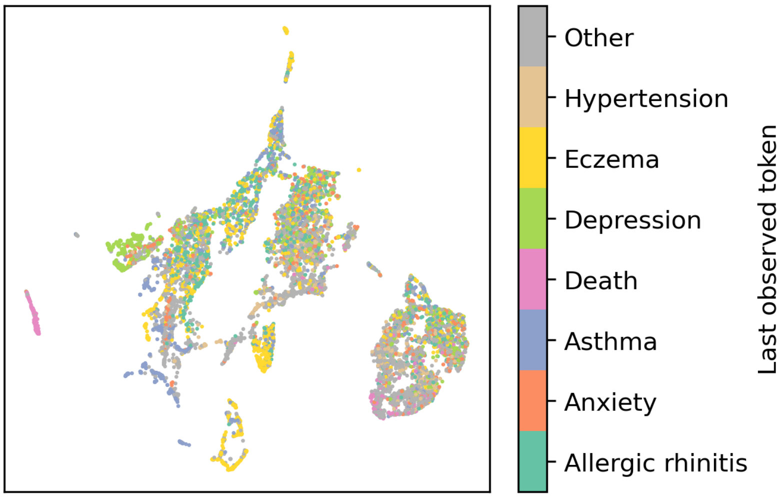

(b) Last observed diagnosis.

Supplementary Figure S4: Learnt representations with baseline covariates masked. UMAP representations $\mathbf{T}_c$ of patient health records in the held-out pre-training cohort with baseline covariates $\mathbf{e}^{\text{static}}$ removed. We include histories up to and including each diagnosis a patient experiences.

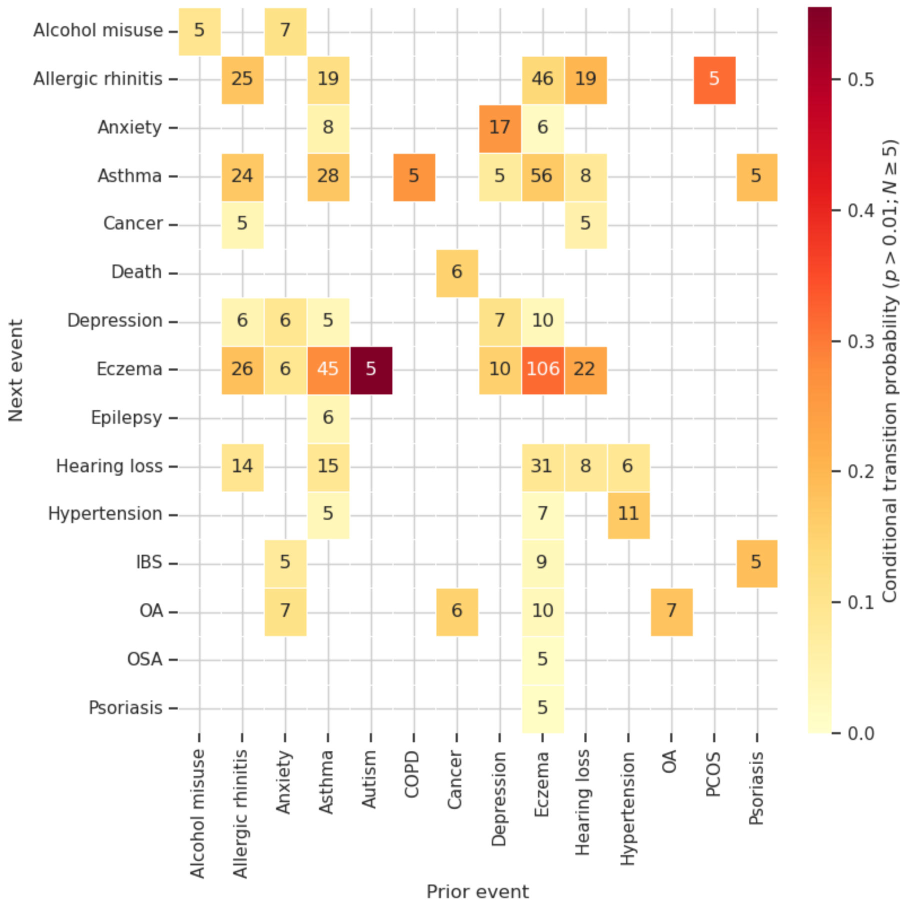

Supplementary Figure S5: Forecasting known diagnosis to diagnosis associations in the pre-training cohort. Given unseen patient histories from the pre-training cohort, we predict the next 3 events a patient may experience. From these we plot the transitional probability matrices for a new diagnosis following a previous diagnosis. Transitions with fewer than 5 occurrences and probability below 0.01 are removed for clarity.

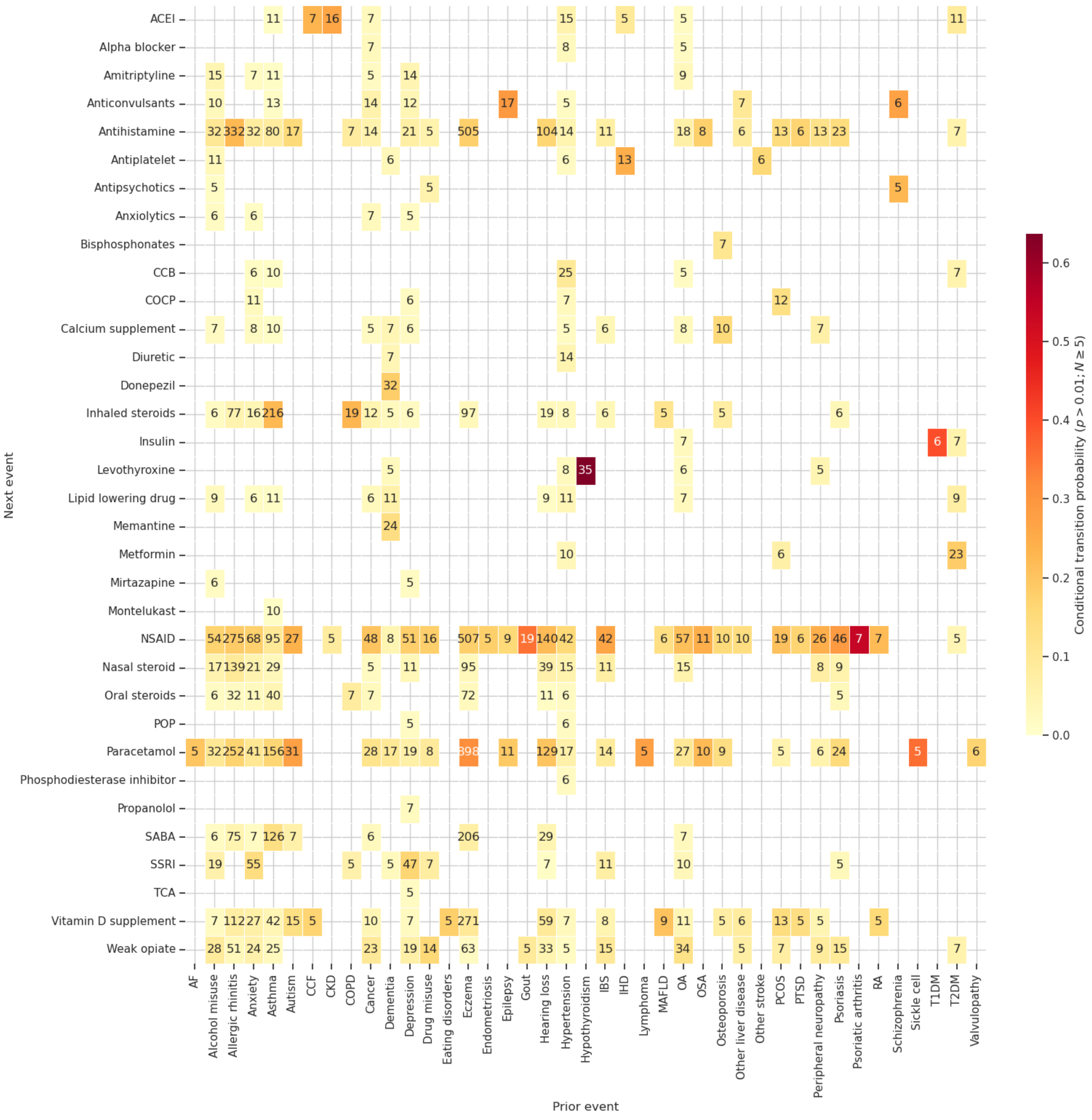

Supplementary Figure S6: Forecasting known diagnosis to medication associations in the pre-training cohort. Given unseen patient histories from the pre-training cohort, we predict the next 3 events a patient may experience. From these we plot the transitional probability matrices for a new medication following a previous diagnosis. Transitions with fewer than 5 occurrences and probability below 0.01 are removed for clarity.

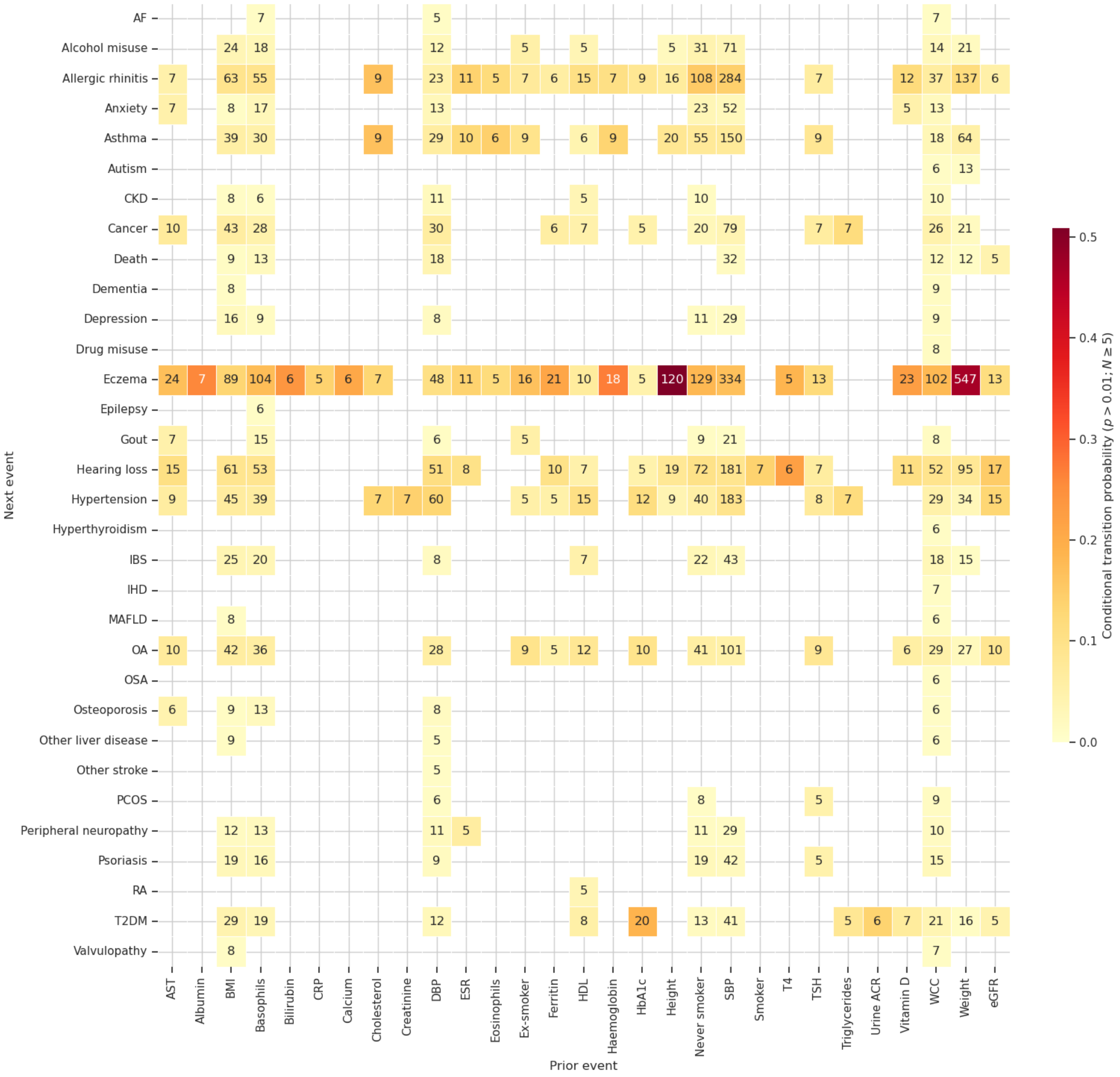

Supplementary Figure S7: Forecasting known investigation to diagnosis associations in the pre-training cohort. Given unseen patient histories from the pre-training cohort, we predict the next 3 events a patient may experience. From these we plot the transitional probability matrices for a new diagnosis following a previous investigation. Transitions with fewer than 5 occurrences and probability below 0.01 are removed for clarity.

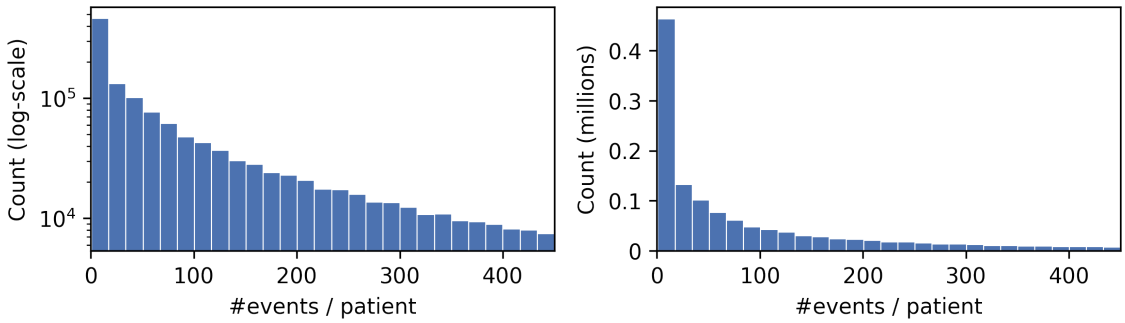

Supplementary Figure S8: Forecasting known investigation to diagnosis associations in the pre-training cohort. Given unseen patient histories from the pre-training cohort, we predict the next 3 events a patient may experience. From these we plot the transitional probability matrices for a new diagnosis following a previous investigation. Transitions with fewer than 5 occurrences and probability below 0.01 are removed for clarity.
